# Direct Observation of the Uptake of Outer Membrane Proteins by the Periplasmic Chaperone Skp

**DOI:** 10.1371/journal.pone.0046068

**Published:** 2012-09-26

**Authors:** Zhi-Xin Lyu, Qiang Shao, Yi Qin Gao, Xin Sheng Zhao

**Affiliations:** 1 State Key Laboratory for Structural Chemistry of Unstable and Stable Species, Department of Chemical Biology, Biodynamic Optical Imaging Center, College of Chemistry and Molecular Engineering, Peking University, Beijing, China; 2 Beijing National Laboratory for Molecular Sciences, Peking University, Beijing, China; 3 Institute of Theoretical and Computational Chemistry, College of Chemistry and Molecular Engineering, Peking University, Beijing, China; University of Cambridge, United Kingdom

## Abstract

The transportation of membrane proteins through the aqueous subcellular space is an important and challenging process. Its molecular mechanism and the associated structural change are poorly understood. Periplasmic chaperones, such as Skp in *Escherichia coli*, play key roles in the transportation and protection of outer membrane proteins (OMPs) in Gram-negative bacteria. The molecular mechanism through which Skp interacts with and protects OMPs remains mysterious. Here, a combined experimental and molecular dynamics simulation study was performed to gain the structural and dynamical information in the process of OMPs and Skp binding. Stopped-flow experiments on site specific mutated and labeled Skp and several OMPs, namely OmpC, the transmembrane domain of OmpA, and OmpF, allowed us to obtain the mechanism of OMP entering the Skp cavity, and molecular dynamics simulations yielded detailed molecular interactions responsible for this process. Both experiment and simulation show that the entrance of OMP into Skp is a highly directional process, which is initiated by the interaction between the N-terminus of OMP and the bottom “tentacle” domain of Skp. The opening of the more flexible tentacle of Skp, the non-specific electrostatic interactions between OMP and Skp, and the constant formation and breaking of salt bridges between Skp and its substrate together allow OMP to enter Skp and gradually “climb” into the Skp cavity in the absence of an external energy supply.

## Introduction

The cell envelope of Gram-negative bacteria is composed of inner membrane, outer membrane, and periplasmic space between them. As the first line of contact between bacteria and their external environment, the outer membrane contains an important class of integral β-barrel outer membrane proteins (OMPs) associated with basic biological functions, virulence, and multidrug resistance [1]–[4]. OMPs, such as OmpA, OmpC, OmpF, maltoporin (LamB), and phosphoporin (PhoE), function as channels spanning the outer membrane and have been identified as adhesins, protein translocation pores, passive diffusion pores, siderophore receptors, as well as proteins of other functions [1], [5]. Blocking the processes involved in the biogenesis of bacterial OMPs impairs their crucial biological functions and therefore tremendously reduces the cellular viability. Because of its biological importance and potential applications on medicine, numerous reviews have been written in recent years on the biogenesis of bacterial OMPs [6]–[12]. Furthermore, besides the Gram-negative bacteria, mitochondria and chloroplasts in eukaryotes are also surrounded by two biological membranes. The biogenesis of the OMPs is considered to be evolutionally conserved and many key proteins involved are homologues [11], [13]–[16]. Currently, relatively little is known about the biogenesis pathways of OMPs in mitochondria and chloroplasts. The mechanism by which OMPs are transported and inserted into the outer membrane of Gram-negative bacteria is expected to be a favored paradigm for mitochondria and chloroplasts in eukaryotes [15], [16].

OMPs are generally synthesized in the cytoplasm. Before their final folding and insertion into the outer membrane, OMPs must translocate across the inner membrane and then transport through the periplasmic space. Research interests evolve around the mysteries of the biogenesis of OMPs, and particular attention has been paid to their long-distance transportation and subsequent folding and insertion into the outer membrane. Experiments demonstrated that three periplasmic proteins, the 17 kDa protein Skp, the survival factor SurA, and the degradation protein DegP, are among the major factors that safeguard the OMP transportation through the periplasmic space, where the major role of Skp and SurA is that of a molecular chaperone. Whether and how DegP exercises its chaperone/protease dual function [17], [18] remain unclear. Our recent study showed that Skp and SurA interact with OMPs much faster than DegP, but on the other hand DegP binds to OMPs more strongly than Skp and SurA [19]. We have also found that *in vivo* Skp and SurA function as chaperones and DegP acts mainly as protease at 37°C. Based on these observations, a kinetic partitioning mechanism was proposed: after being secreted from the inner membrane into the periplasmic space OMPs are immediately safeguarded by Skp and SurA, while DegP works downstream to either clean up misfolded OMPs or help OMPs on their journey to the outer membrane, depending on whether OMPs are appropriately protected by Skp/SurA. The following questions are raised naturally. How do Skp and SurA interact with OMPs quickly? What are the detailed molecular mechanisms for the interactions of OMPs with Skp, SurA, and DegP? This article is devised to address these questions with regard to Skp.

In aqueous solutions, three monomers of Skp associate to form a stable trimer with a “jellyfish” architecture [20], [21]. Each monomer of Skp consists of 141 residues and adopts predominantly a continuous α-helical secondary structure (∼80%) with a β-sheet at the termini. The β-sheets in the monomers assemble together to form a β-barrel core domain at the top of the trimer. The α-helical structures, which are also called the tentacle domains, protrude about 60 Å from the core domain, resulting in a central cavity surrounded by three tentacles. The tentacles have a side-to-side distance of about 25 Å. Circular dichroism (CD) and fluorescence spectroscopy experiments of Bulieris et al. showed that OmpA can bind the Skp trimer to form a stable complex at a 1∶1 stoichiometry at pH 7.0, which efficiently prevents aggregation of OmpA in solutions [22]. Other OMPs, such as OmpG and YaeT, were reported to form 1∶1 complexes with Skp in the recent fluorescence spectroscopy experiment of Qu et al. [23]. Moreover, by measuring the disulfide cross-linking of Skp to OmpA, Walton et al. observed that in the Skp-OmpA complex, the transmembrane β-barrel domain of OmpA is inside the cavity of Skp whereas the soluble periplasmic domain stays outside [24]. Based on these experimental observations, it is reasonable to postulate that as Skp binds an OMP, it holds OMP in its central cavity [20]. However, the mechanism for OMP to enter the cavity of Skp remains obscure given the large size of OMP.

**Figure 1:**
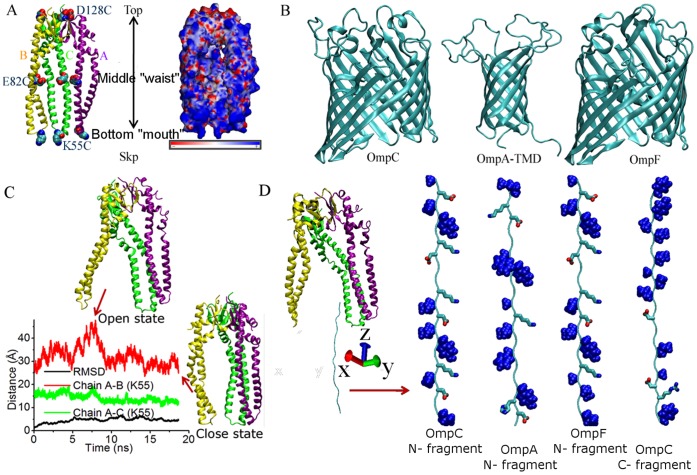
The structures of Skp and outer membrane proteins used in experiments and simulations. (A) The crystal structure of Skp (chain A: purple, chain B: yellow, and chain C: green, PDB code: 1SG2 [21]) and its electrostatic potential. Residues replaced by cysteine in experiments are shown using the VDW mode. (B) The monomeric crystal structures of OmpC, the transmembrane domain of OmpA, and OmpF (PDB codes: 2J1N, 2GE4, and 2OMF [41]–[43]). (C) Time series of the RMSD value of Skp with respect to its crystal structure, and distances between the Lys55 residues in chains A and B (red) and in chains A and C (green) of Skp in water from MD simulations. (D) The simulation system of Skp and OMP polypeptides in water. In each polypeptide, hydrophobic residues are shown with the VDW mode and charged ones are shown using the licorice mode.

In the present article, we report results from a combined experimental and simulation study, using stopped-flow kinetics measurements and molecular dynamics (MD) simulations to gain insights on how three OMPs (OmpC, OmpA, and OmpF) enter the central cavity of Skp. No cysteine residue exists in both the wild-type sequences of Skp and OmpC. In the experiment, cysteine residues were introduced at selected sites of Skp and OmpC. For Skp, they were K55C, E82C, and D128C, corresponding to the bottom “mouth”, middle “waist”, and top positions, respectively (Figure 1A). Since Skp forms a trimer in the aqueous solution, replacement of each of the three abovementioned residues by cysteine results in the triple occupation of cysteine at symmetric positions in the three identical tentacles. As revealed previously by various experiments, self-quenching is a common photophysical phenomenon caused by the close contact between the dyes [25]–[30]. Therefore, by measuring the fluorescence intensity of Cy3 introduced in the three Skp mutants, the stopped-flow technique can be used to track distance changes of the Cy3 dyes labeled at selected sites during the entrance of the urea-denatured OMP into Skp. Similar experiments with fluorescence resonance energy transfer (FRET) between Skp and different OmpC mutants (D25C, L139C, and D290C, respectively) as indicators for the distances between various positions of Skp and OmpC are also conducted. These measurements give direct evidence that OMPs enter the Skp cavity through the open bottom of its tentacles, initiated from the N-terminus of OMPs. Meanwhile, MD simulations are performed to investigate interactions between OMP (segments of OMPs were used in the simulations to reduce computational costs) and Skp at the atomic level. The consistency in the experimental and simulation results leads to a more concrete and molecular detailed understanding of interactions between OMP and Skp and the entrance of OMP into the cavity of Skp.

## Results

### Structural Stability of Skp in Water

The “jellyfish” architecture of Skp is shown in Figure 1A. The on-membrane monomer models of OmpC, the transmembrane domain of OmpA (OmpA-TMD), and OmpF are shown in Figure 1B. The electrostatic potential of Skp calculated using AMBER’s MM_PBSA (molecular mechanics-Poisson-Bolzmann/surface area) module [31] shows that while the core domain at the top of Skp is largely negatively charged, the entire surface of tentacle domains (particularly the bottom of tentacles) is rich in positive charges **(** Figure 1A). In fact, Skp is a very basic protein with a pI value around 10.5 [32]. To check the structure stability of Skp in the aqueous solution, we ran MD simulations on Skp in water and the calculated trajectory is shown in Figure 1C. The initial atomic coordinates of Skp were taken from its crystal structure, of which the tentacle bottoms of chains A and C are in contact while the bottom of Chain B is crystallographically disordered [21]. This missing segment of chain B was added in our study by aligning it to chains A and C. In the simulation time of ∼20 ns, the overall root-mean-square deviation (RMSD) of Skp in comparison with its initial structure fluctuates within 7 Å. The variation of the structure is mainly caused by the flexible bottom domain of chain B (RMSD_chainB_ goes to ∼4.7 Å at the end of the simulation). The rest structure of Skp remains stable (RMSD_chainA_, RMSD_chainC_ ≤3.0 Å). As shown in Figure 1C, the distance between the Lys55 residues located in the bottom of chains A and C fluctuates within a very small range while that between chains A and B varies significantly during the simulation. Therefore, in the simulation, chains A and C remain in tight contact with each other in water whereas the bottom of chain B flops back and forth with respect to the other two chains. Chain B being considerably more flexible compared to the other two is consistent with the difficulties in obtaining its X-ray structure [21].

### OMP Enters Skp from the Bottom of Skp

**Figure 2:**
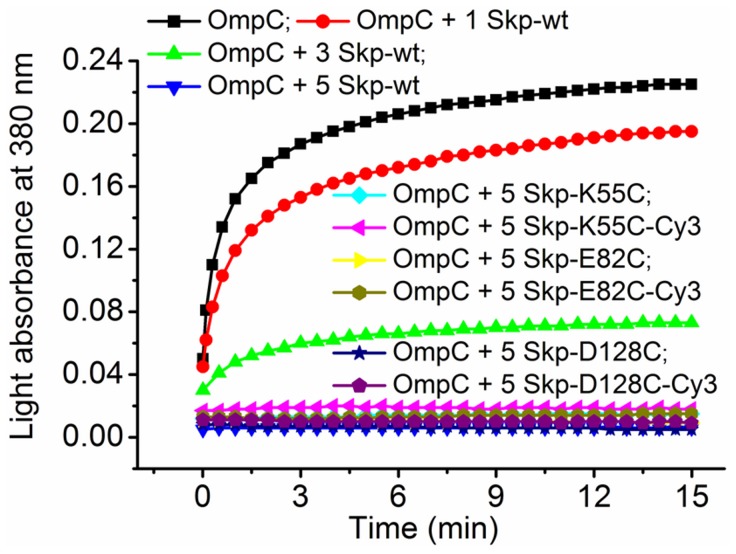
**Verification of the chaperone activity of Skp mutants.** Urea-denatured OmpC was diluted to a final concentration of 1.5 µM into PBS buffer alone or containing various concentrations of wild-type Skp (1.5 µM, 4.5 µM and 7.5 µM) and 5-fold molar excess of labeled or unlabeled Skp mutants (7.5 µM) at 25°C. Aggregation of OmpC was monitored by the apparent absorbance increase at 380 nm caused by light scatter.

**Figure 3:**
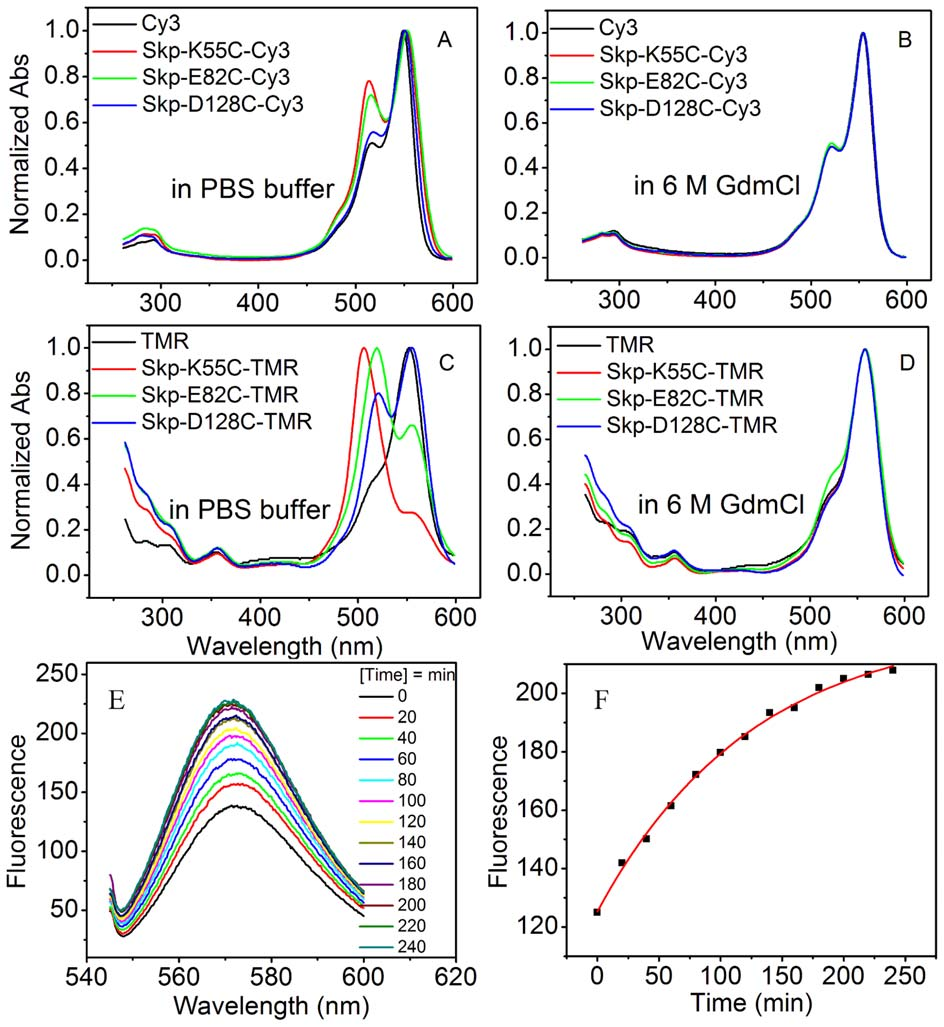
**Absorption spectra of fluorescently labeled Skp mutants.** (A*–*B) Absorption spectra of Cy3 labeled Skp mutants were collected in 50 mM PBS buffer and buffer containing 6 M GdmCl at pH 7.0, respectively. (C*–*D*)* Absorption spectra of TMR labeled Skp mutants were collected in 50 mM PBS buffer and buffer containing 6 M GdmCl at pH 7.0, respectively. Samples were incubated in 6 M Gdmcl buffer at 37°C for 1 h to denature the proteins. (E) Fluorescence emission of Cy3 dye was monitored immediately after 5-fold molar excess of unlabeled Skp-wt was added into the Skp-E82C-Cy3 solution with an interval of 20 min at 37°C. (F) The change of fluorescence intensity via time was fitted to a single exponential, with a half time t_1/2_ = 128 s.

For the Cy3 dye labeled Skp mutants (Skp-K55C-Cy3, Skp-E82C-Cy3, and Skp-D128C-Cy3), measurements of far-UV CD spectroscopy and aggregation assays (Figure S1 and Figure 2) showed that the protein modifications and dye labeling did not perturb the secondary structure or the chaperone activity of Skp. Meanwhile, the UV-Vis absorption spectral line shapes and peak locations of Cy3 on Skp are significantly different from those of the isolated Cy3 dye (Figure 3A). The spectral change is especially prominent for Skp-K55C-Cy3 and Skp-E82C-Cy3, indicating the existence of the dye self-quenching at these sites. TMR, a fluorescent dye proved to be very sensitive to microenvironment changes, was also used to label the same three sites of Skp and the absorption spectra showed stronger variations, confirming the self-quenching phenomenon (Figure 3C). In addition, the protein denaturation is expected to separate the dyes labeled on proteins and thus can eliminate fluorescence self-quenching. The fact that absorption spectra of Cy3 or TMR labeled Skp mutants denatured in 6 M GdmCl solution showed no differences among themselves or from the isolated dyes (Figure 3B and 3D) again supports the self-quenching mechanism. Self-quenching among Cy3 dyes in Skp trimer was further confirmed by subunit exchange experiments. By mixing Skp-E82C-Cy3 with 5-fold molar excess of unlabeled Skp (Skp-wt), the subunit exchange between them reduced the molecular number of paired Skp-E82C-Cy3. As a result, the fluorescence emission of Cy3 dye is expected to increase with time, as indeed observed in the experiments (Figure 3E and 3F).

Next, fluorescence correlation spectroscopy (FCS) experiments were performed to measure the diffusion time (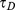) of each Skp mutant bound to OmpC-Cy3 and the isolated Cy3 dye (Figure S2). The molecular weight of each Skp-OmpC complex was then evaluated using individual Cy3 dye as the reference (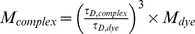), and the calculated result was 85.8±2.6 kD for Skp-wt/OmpC, 82.6±2.5 kD for Skp-K55C/OmpC, 87.5±2.6 kD for Skp-E82/OmpC, and 90.3±2.7 kD for Skp-D128C/OmpC, respectively. The molecular weights of these complexes match the sum of weights of OmpC (∼39 kD) and Skp mutant (∼50 kD), indicating that Skp mutants form 1∶1 complexes with OmpC.

**Figure 4:**
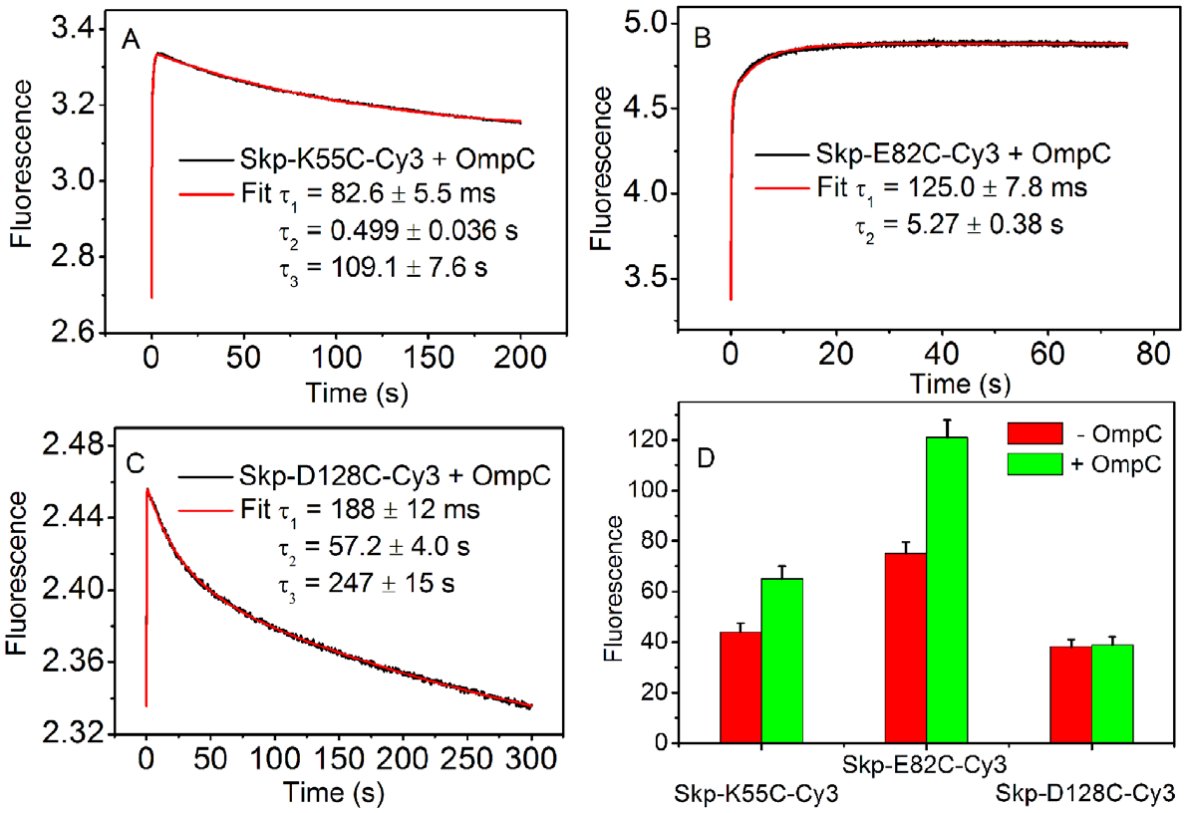
**Binding of OmpC with Cy3 labeled Skp mutants measured by stopped-flow kinetics experiment.** (A*–*C) Fluorescence time-course monitoring the entrance of OmpC into the Cy3 dye labeled Skp mutants (Skp-K55C-Cy3, Skp-E82C-Cy3, and Skp-D128C-Cy3). The data were fitted to functions containing two- or three- exponential terms. (D) Comparison of the static fluorescence intensity of Cy3 dye labeled Skp mutants in the presence and absence of OmpC.

Figure 4 shows the traces of fluorescence intensity of Cy3 after Skp-Cy3 was mixed with OmpC, measured by stopped-flow kinetics experiment. In these experiments, OmpC was denatured in 8 M urea before the mixing and was mixed with Skp-Cy3 mutants with a volume ratio of 1∶20, so that the urea concentration was diluted to 0.38 M. The final concentrations of Skp and OmpC were both 0.03 µM. The time-courses were triphasic at K55C and D128C sites and biphasic at E82C, which were well described by three- and two-exponential processes, respectively. The first phase is accompanied by a sharp increase of fluorescence intensity observed in all curves with characteristic times consistent with previous measurements based on different labeling schemes [19]. The sharp increase of fluorescence intensity at the early time suggests that the Cy3-to-Cy3 distance increases when OmpC goes through the labeling sites. The fitted time constants for this phase, *τ*_1_, increase in the order of Skp-K55C-Cy3 (82.6±5.5 ms), Skp-E82C-Cy3 (125.0±7.8 ms), and Skp-D128C-Cy3 (188±12 ms), suggesting that Skp “swallows” OmpC through its “mouth” and OmpC “climbs” up into the Skp cavity (Figure 4A, B, C). In order to quantitatively fit the changes of fluorescence intensity, a slower second phase was needed in each curve. The time constants, *τ*_2_, of the second phase are within the range of 0.5–60 s, suggesting that there exist other dynamical processes such as finer conformational rearrangements of the Skp-OmpC complex during OmpC enters Skp. Finally, the third and slowest decrease phase (*τ*_3_>100 s) was observed for K55C and D128C, indicating that the dye-to-dye distance reduced later at respective labeling sites. The measurement on static fluorescence of Skp-Cy3 in the presence and absence of OmpC (Figure 4D) confirmed the results on the final fluorescence intensities in the stopped-flow measurements (Figure 4A, B, C).

It is intriguing to make a “movie” for Skp and OMP binding using the experimental data. For K55C, the fluorescence intensity first increased and then dropped later on, but to a value still higher than that before Skp started to “swallow” OmpC, consistent with a picture that Skp opened the “mouth” to “swallow” OmpC and afterwards closed its “mouth” but only partially, because OmpC had entered the cavity. For E82C, the increase of fluorescence intensity occurred later than K55C and lacked a decreasing phase, indicating that the Skp “waist” met OmpC later than the “mouth” and the cavity kept expanding in the entire process of OMP entrance. For D128C, the change of fluorescence intensity was the slowest and transient: it first increased and, after the reaction, recovered completely to its initial level, indicating that the top part of Skp was affected by the entrance of OmpC into the cavity at last and the transient disturbance on this part disappeared after OmpC settled down in the Skp cavity.

Next, we performed similar experiments on Skp-Cy3 with OmpA-TMD and Skp-Cy3 with OmpF. These results resembled that of OmpC (Figures S3 and S4) and generated exactly the same physical picture. The observed *τ*_1_ values at the three Cy3 dye labeled sites followed the same order, i.e., it increased as the labeling site moved from the bottom to the top of Skp, showing that all OMPs under study enter the cavity of Skp from its “mouth”. In addition, the *τ*_1_ values for Skp-OmpF (61.3±4.9 ms for Skp-K55C-Cy3, 182±11 ms for Skp-E82C-Cy3, and 270±16 ms for Skp-D128C-Cy3) are similar to that for Skp-OmpC, presumably because OmpF and OmpC have the similar molecular weight. The *τ*_1_ values for Skp-OmpA (38.6±2.7 ms for Skp-K55C-Cy3, 61.7±4.2 ms for Skp-E82C-Cy3, and 104.7±7.3 ms for Skp-D128C-Cy3) are apparently smaller than the corresponding quantities for Skp-OmpC and Skp-OmpF. The magnitudes in the change of the fluorescence intensity of Skp-OmpA (Figure S3) are also smaller than that of Skp-OmpC and Skp-OmpF (Figures 4 and S4). Both observations are consistent with the fact that OmpA has a smaller size compared to the other two OMPs.

### The N-terminus of OMP Enters Skp First

**Figure 5:**
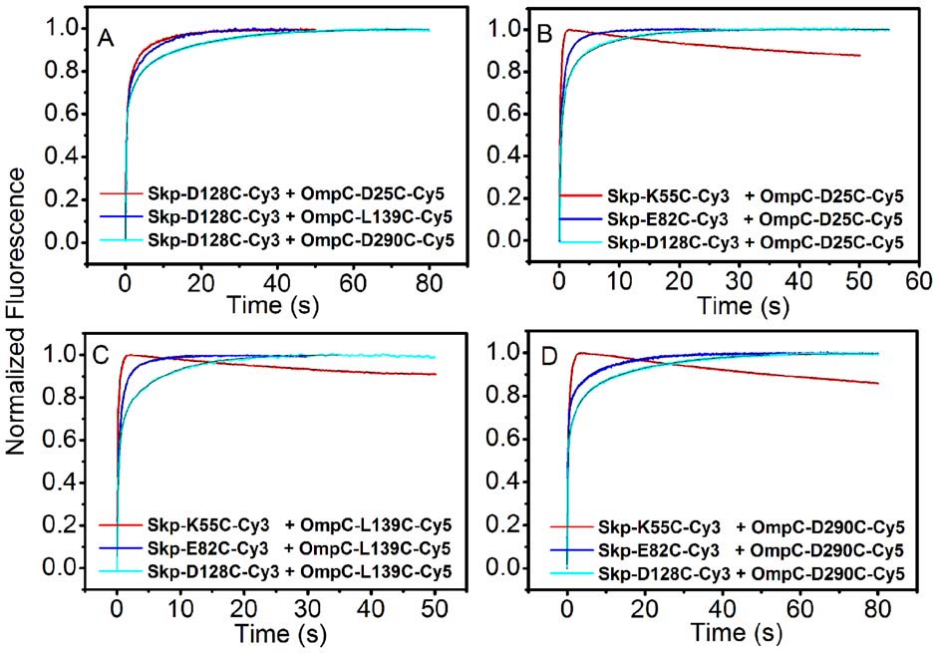
Binding of Cy3 labeled Skp mutants with Cy5 labeled OmpC mutants measured by FRET based stopped-flow kinetics experiment. (A) Fluorescence time-course monitoring the entrance of three Cy5 dye labeled OmpC mutants into Skp-D128C-Cy3. (B*–*D) Fluorescence time-course monitoring the entrance of each of three Cy5 dye labeled OmpC mutants into the three Cy3 dye labeled Skp mutants.

To gain further insight into how OmpC enters Skp, we introduced cysteine on both Skp and OmpC at specific sites and performed FRET based stopped-flow kinetics experiment. The acceptor (Cy5) fluorophore was attached to OmpC at positions D25C, L139C, and D290C, respectively, along the polypeptide chain from the N- to C-terminus and thus generated three mutants (OmpC-D25C-Cy5, OmpC-L139C-Cy5, and OmpC-D290C-Cy5). The donor (Cy3) fluorophore was attached to each chain of Skp at also three different positions (K55C, E82C, and D128C), respectively, as described earlier. As shown in Figure 5A, when Skp-D128C-Cy3 was mixed with each of the Cy5 dye labeled OmpC mutants at a 1∶1 ratio, the fluorescence emission from the acceptor (Cy5) fluorophore increased dramatically, corresponding to the approach of the acceptor to the donor while OmpC enters the cavity of Skp. All of the traces were well fitted to triple exponentials (the necessity of triple exponential fitting is illustrated in Figure S5). The data clearly demonstrate that the N-terminus of OmpC moves into the Skp cavity first. The fitted time constants of this fast phase *τ*_1_ are 143±10 ms, 208±14 ms, and 285±19 ms for the Cy5 dye labeled sites of D25C, L139C, and D290C in OmpC, respectively (Table S2). It is apparent that the time constant increases as mutated residues moving from N- to C-terminus of OmpC. As shown in Table S2, the same ranking order of the time constants was also observed for the Cy5 dye labeled OmpC mutants mixed with either Skp-K55C-Cy3 or Skp-E82C-Cy3. These results suggest that the N-terminus of OmpC (D25C) enters the cavity of Skp prior to its C-terminus (D128C).

On the other hand, for the three Cy3 dye labeled Skp mutants mixed with OmpC-D25C-Cy5, a sharp increase of Cy5 signal was observed for each of the three mutation sites of Skp, with the fitted time constant of 73.0±5.1 ms for Skp-K55C-Cy3, 101.3±6.9 ms for Skp-E82C-Cy3, and 143.0±10.0 ms for Skp-D128C-Cy3, respectively (Figure 5B and Table S2). The time constants increase as the labeling sites move from the bottom to top of Skp. The same order was also observed for the three Cy3 dye labeled Skp mutants mixed with either OmpC-L139C-Cy5 or OmpC-D290C-Cy5 (Figure 5C and D, and Table S2). These results again demonstrate that OmpC enters Skp from the bottom to the top of Skp, consistent with the stopped-flow experiments on the mixture of the three Cy3 dye labeled Skp mutants and unlabeled OmpC (Figure 4). It is interesting to notice that the FRET data shows different aspects of the site-to-site distances from the self-quenching data during OMPs enter the Skp cavity, although they both characterize the reaction rates. Figure 5B, C, D show that the distance from each residue in OmpC to the bottom of Skp first shortened and then elongated. However, the distance from each residue of OmpC to the waist and the top of Skp never decreased, firmly showing that OMPs went through the bottom and entered completely into the Skp cavity (OmpC has 346 residues and the last labeled site is the 290^th^ one).

### Molecular Details of the Entrance of OMP into Skp

MD simulations were performed to obtain information on the molecular-detailed interactions between OMP and Skp. The selected OMPs include OmpC, OmpA, and OmpF in accordance with the experiments. Guided by the experimental findings and to reduce computational cost, we used short polypeptides cut from the N- or C- terminus of OMPs instead of the full length membrane proteins. Urea-denatured OMPs have large gyration radii and require a large system for simulation. The computational cost on the simulation of binding of the complete OMP structure to Skp is very high.

**Figure 6:**
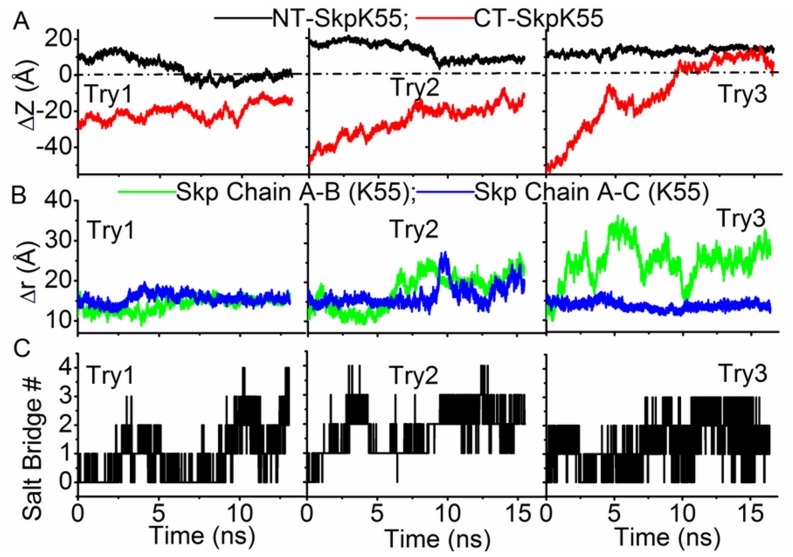
Interactions of N-terminal fragment of OmpC with closed Skp. (A) Time evolution of the distance in the z-direction between Lys55 of Skp and the N-terminal residue (NT) or C-terminal residue (CT) of the N-terminal fragment of OmpC when the closed state of Skp is used in simulation. (B) Time evolution of distances between the Lys55 residues in three chains of Skp. (C) Time evolution of the salt bridge number formed between the N-terminal fragment of OmpC and Skp.

Two states of Skp, which are referred as “open” and “closed” states and have the bottom of Skp open and closed respectively, were used as the initial structures of Skp in the simulations (both structures of Skp were observed in the simulation trajectory of Skp in water, see Figure 1C). We first performed the simulation starting with the closed state of Skp and the N-terminal polypeptide of OmpC (sequence: AEVYNKDGNKLDLYGKVDGL). An extended structure of the N-terminal polypeptide was placed initially with its N-terminal residue (Ala1) adjacent to the bottom of Skp, as shown in Figure 1D. Three typical trajectories of independent simulations are shown in Figure 6. Figure 6A shows the time evolution of the distance in the z-direction (ΔZ) between each of the two terminal residues of the OmpC polypeptide (Ala1 or Leu20) and Lys55 of Skp. Positive ΔZ corresponds to the polypeptide residue being above Lys55 and thus inside the Skp cavity. ΔZ is negative when the residue is outside of Skp. In the first and second trajectories, the N-terminal residue of the OmpC polypeptide which was placed initially inside Skp showed a tendency of leaving Skp, as revealed by the decrease of its ΔZ value. In the meanwhile, although the bottom end (C-terminal residue) of the polypeptide approached closer to Skp as a result of structural collapse of the polypeptide, it remained outside of Skp (ΔZ<0) during the simulation. At the final stage of these two simulations, the polypeptide adhered to the bottom of Skp by forming salt bridges. The calculated number of salt bridges between the polypeptide and Skp is shown in Figure 6C. In the third trajectory, the N-terminal residue of the polypeptide remained in Skp whereas the C-terminal residue moved gradually into Skp (ΔZ>0). It is interesting to note that in the first two trajectories the three chains of Skp remained in the closed state whereas in the last trajectory chain B moved away from A and C chains and as a result Skp converted to the open state (Figure 6B). These results indicate that the opening of Skp is essential for the entrance of OMP.

**Figure 7:**
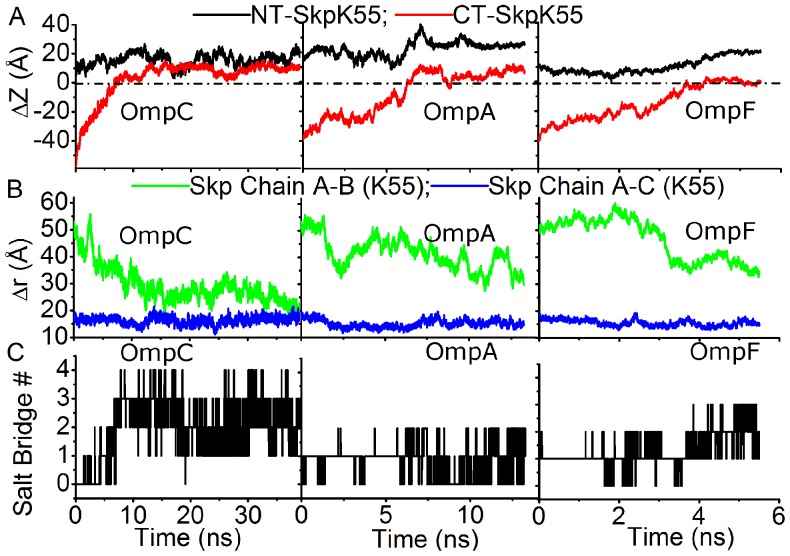
**Interactions of N-terminal fragments of OMPs with opened Skp.** (A) Time evolution of the z-direction distance between Lys55 of Skp to the N-terminal residue (NT) and the C-terminal residue (CT) of N-terminal fragments of OmpC, OmpA, and OmpF in the corresponding trajectories with open state of Skp respectively. (B) Time evolution of distances between the Lys55 residues in three chains of Skp. (C) Time evolution of the salt bridge number formed between the N-terminal fragment of OmpC and Skp.

Next, we then tried a number of simulations starting from the open state of Skp. Three polypeptides corresponding to the N-terminal fragments of OmpC, OmpA (sequence: APKDNTWYTGAKLGFSQYHDT), and OmpF (sequence: AEIYNKDGNKVDLYGKAVGL), respectively, were used in these simulations. It is easy to see from Figure 7A that all three polypeptides successfully translocated into Skp: the N-terminus of each polypeptide moved further into the Skp cavity (compared to the initial position, the top end of the polypeptide moved up by ∼17.5 Å for OmpC, ∼12 Å for OmpA, and ∼10 Å for OmpF) and the bottom end also inserted into Skp. In the meanwhile, distances between the strayed chain B and other two chains of Skp decreased gradually in all three simulations (Figure 7B). Therefore the bottom of the three chains of Skp became closed after the polypeptide moved into Skp.

Combining the results from abovementioned simulations, we suggest that the opening of Skp is one of the main determinants for the successful entrance of polypeptides into Skp, and Skp changes towards the closed state after the polypeptide moves into its cavity, due to the interactions among Skp subunits and the enclosed polypeptide. This conformational change of Skp after taking-up OMP polypeptides is consistent with the experimental observation of the third phase of Skp-K55C mutant in the stopped flow measurement.

**Figure 8:**
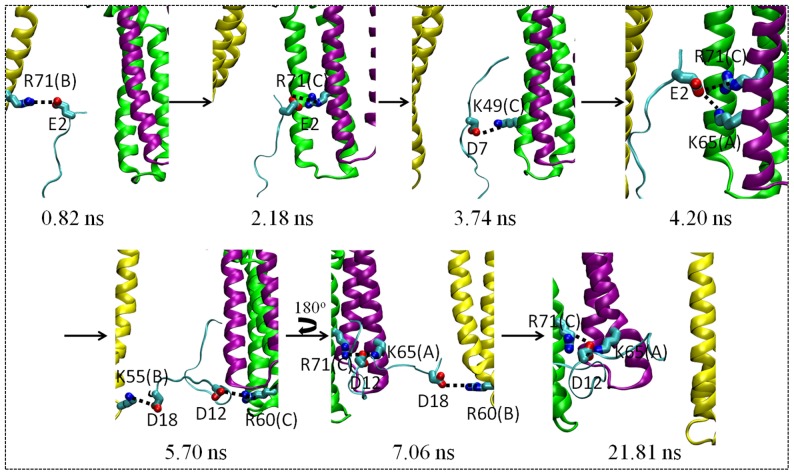
**Snapshots from the simulation trajectory of N-terminal fragment of OmpC in the bottom of open Skp.** Residues involved in forming salt bridges between the polypeptide and Skp are shown using the licorice mode. For each Skp residue, the corresponding chain is recorded in the parentheses.

As discussed in the following, the other important factor for various polypeptides to penetrate into open Skp (Figure 7) as well as to prevent them from retreating from Skp (Figure 6) is the electrostatic interactions between the polypeptides and Skp. Skp and polypeptides can form multiple salt bridges between them, as shown in Figures 6C and 7C. In what follows, we use the N-terminal fragment of OmpC as an example to illustrate the process that a polypeptide moves into open Skp aided by salt bridges. This OmpC N-terminal polypeptide contains four negatively charged residues (Glu2, Asp7, Asp12, and Asp18) and three positively charged ones (Lys6, Lys10, and Lys16). Examples of snapshots taken from the simulation trajectory of this polypeptide and Skp in water (left panel in Figure 7A) are given in Figure 8. The movement of the polypeptide into Skp was accompanied by the breaking and formation of a series of salt bridges between the polypeptide and Skp: Glu2–Arg71 (chain B) → Glu2–Arg71 (chain C) → Asp7–Lys49 (chain C) → Glu2–Lys65 (chain A), Glu2–Arg71 (chain C) → Asp12–Arg60 (chain C), Asp18–Lys55 (chain B) → Glu12–Arg71 (chain C), Glu12–Lys65 (chain A), Asp18–Arg60 (chain B). The polypeptide therefore “climbs” along the inside wall of Skp under the aid of salt bridges formed between them.

Similar to OmpC, the N-terminal fragment of OmpA contains two negatively and three positively charged and that of OmpF has three negatively and three positively charged residues. The translocation of these two polypeptides on the inside wall of Skp and thus the successful entrance of these two polypeptides into Skp is also facilitated by the salt bridge formation, which is clearly seen from the snapshots from the corresponding trajectories in Figures S6 and S7, with the following order of salt bridge breaking and formation: D4–K65 (chain A) → D4–K65 (chain A), D20–K49 (chain C) → D4–R71 (chain B), D20–K49 (chain C) → K3–E82 (chain C), D20–K49 (chain C) for OmpA, and E2–R71 (chain C) → E2–R71 (chain C), D7–K55 (chain B) → E2–R71 (chain C), E2–R91 (chain A), D7–K55 (chain B) for OmpF.

The simulations discussed above showed that the OMP polypeptides could enter Skp from the bottom. To examine the possibility of polypeptides entering Skp from other positions, two trajectories for the N-terminal fragment of OmpA and one trajectory for the N-terminal fragment of OmpC were run with the polypeptides placed initially at the side of Skp (Figure S8). At the beginning of each trajectory, the polypeptide was placed vertical to the symmetry axis of Skp. The distance between the first residue of the polypeptide and the E82 residue in the middle of chain A in Skp is then used to characterize the movement of the polypeptide relative to Skp. In the simulation, the OmpC polypeptide moved away from Skp, as revealed by the increasing distance between the defined residues as above in Figure S8. In its first trajectory, the OmpA N-terminal polypeptide moved away from Skp and in the second one it remained adhered to the outside wall of Skp. These simulations showed that the polypeptides, at least in the time scale of simulations, are not capable of entering Skp from its side.

As negative control, simulations were performed using four uncharged polypeptides, namely, the N-terminal fragment of OmpC (OmpA) with all charged residues replaced by Alanines (OmpC Ala mutant, OmpA Ala mutant) and the N-terminal fragment of OmpC (OmpA) with all charged residues replaced by Asparagines (OmpC Asn mutant, OmpA Asn mutant). Figures S9 and S10 show that after ∼20 ns simulation time all of the four polypeptides moved out of Skp, indicating that without salt bridges formed between them, OMP could not enter Skp.

In summary, the stopped-flow experiments demonstrated that as OMP binds to Skp, it enters the central cavity of Skp from the bottom. This conclusion is strongly supported by MD simulations. In the simulations, the OMP N-terminal polypeptides could move into Skp only from the open bottom but not from its side. Moreover, MD simulations provided information on molecular-detailed interactions between OMP and Skp in the entrance process. Electrostatic interaction between OMP and Skp appears to be the dominant driving force for the entrance of OMP into Skp. Salt bridges constantly form and break between OMP and Skp and during this overall directional process OMP climbs up the inner wall of Skp, in a manner as if Spiderman climbs up buildings.

### The C-terminus does not Initiate the Entrance of OmpC into Skp

In the following simulations, the C-terminal fragment of OmpC was first placed with its C-terminus near the bottom of Skp. In contrast to the apparent motion of the N-terminal fragment of OmpC towards the central cavity of Skp, its C-terminal fragment escaped from Skp, as shown by all nine independent trajectories in Figure S11. Analyses were then performed to understand the failure of the C-terminal polypeptide in entering Skp. It was found that, very different from the N-terminal polypeptide, there was at most one salt bridge formed between the C-terminal polypeptide and Skp in each of the nine trajectories (Figure S12). Due to the lack of charges in the C-terminal polypeptide, the sequential formation and breaking of salt bridges between different pairs of residues did not occur between the polypeptide and Skp. The different behavior between the N- and C-termini again indicates the importance of salt bridges formed between the polypeptides and Skp in directing the former into the latter. Based on these results, we speculate that the observed pattern of OmpC entering Skp from its N- to C-terminus is resulted from the difference in electronegativity between the two termini: the N-terminal fragment of OmpC contains eight hydrophobic residues and seven charged residues (Glu2, Lys6, Asp7, Lys10, Asp12, Lys16 and Asp18), whereas the C-terminal fragment contains eleven hydrophobic resides and only three charged ones (Arg3, Asp4, and Asp10). Different electronegativities result in different behaviors of the two polypeptides in interacting with Skp in MD simulations as discussed above.

The characteristics of the polypeptides used in molecular dynamics simulations in the present study (e.g., the sequence length, the number of hydrophobic residues, and the number of charged residues) were collected and analyzed (Table S5). From the table, we could see easily that for all polypeptides which have the similar length, the polypeptides which possess more charged residues (the N-terminal fragments of OmpC, OmpA, and OmpF) could enter Skp whereas those with few charged residues (the C-terminal fragment of OmpC, the N-terminal fragments of OmpC and OmpA with all charged residues mutated by either Alanines or Asparagines) could not.

## Discussion and Conclusions

The entrance of OMP into the central cavity of Skp, the configuration of OMP adopted within Skp, the release of OMP from Skp, and its folding and insertion into the outer membrane are among the most interesting steps in understanding protein-protein interactions between OMP and Skp. The present article focuses on illustrating the first step. To gain a molecular detailed description on how OMP enters Skp, we performed both stopped-flow experiments and MD simulations. By detecting the distance changes of the Cy3 dyes labeled Skp at different positions (bottom, K55C; middle, E82C; and top, D128C) in the process of OMP entering Skp, stopped-flow kinetics experiments showed directly that three OMPs (OmpC, OmpA-TMD, and OmpF) enter the central cavity of Skp from the bottom tentacle domain and move gradually towards the top of Skp. Moreover, FRET-based stopped-flow kinetics experiment showed that the entrance of OmpC into Skp is initiated from the N-terminus of the membrane protein.

All of these experimental observations are in good agreement with results of concurrent MD simulations. In these simulations, only N-terminal fragments of OMPs entered the central cavity of Skp whereas the entrance of the C-terminal fragment did not occur. In addition, the N-terminal fragments of OMPs entered Skp only from the bottom of Skp but not from its side. Detailed analyses of molecular interactions between Skp and OMP polypeptides during the entrance process in MD simulations indicated that the opening of Skp and electrostatic interactions between Skp and OMP are two essential factors for the successful capture of OMP by Skp. The opening of Skp creates an access for OMP, and the alternate salt bridge formation between Skp and OMP is used by OMP to climb along the inner wall of Skp and thus to move into Skp.

Skp is a very basic protein and positively charged (pI ∼ 10.5 [32]) while OMPs of *E. coli* are negatively charged (pI ranging from 4.5 to 6 [23]). The importance of electrostatic interactions in the entrance process of OMP into Skp in the present study is consistent with previous experimental observation by Qu et al. They have found that the binding of OMPs to Skp is pH-dependent and the binding is not observed at very acidic or basic pH, at which OMPs or Skp is neutralized respectively [23].

## Materials and Methods

### Protein Expression, Purification, and Mutagenesis

The pET28a vector carrying the His_6_-tagged Skp, OmpC, OmpA-TMD, and OmpF were transformed into *E. coli* strain BL21 (DE3) cells. Cells were grown in 2 L of LB medium containing 50 µg/mL kanamycin at 37°C, and protein expression was induced with 0.5 mM IPTG at OD_600_ = 0.6. The cultures were grown for an additional 4 h at 37°C and harvested by centrifugation. The cells were pelleted, resuspended in 70 mL of buffer A (50 mM Tris (pH 8.0), 500 mM NaCl and 10mM imidazole) and lysed by ultrasonication. The cytoplasmic fractiob and cell debris were removed by centrifugation at 20000 g for 30 min, and the supernatant was loaded on a Ni-NTA column pre-equilibrated with buffer A. The column was washed extensively with buffer A, and proteins were eluted with buffer B (50 mM Tris (pH 8.0), 500 mM NaCl and 250 mM imidazole). Fractions containing the protein samples were pooled and dialyzed against buffer C (50 mM PB (pH 7.0) and 100 mM NaCl). For the purification of OmpC, OmpA-TMD, and OmpF, 8 M urea was included in buffers A–C referred above in order to keep them in the unfolded state. The purities of proteins were assessed by SDS-PAGE.

The site-directed cysteine mutants of Skp and OmpC were constructed by using Fast Mutagenesis System (TransGen. Biotech) with the plasmids pET28a-*skp* and pET28a-*ompC* as templates (see Table S1 for a summary of Skp and OmpC mutants). The mutated plasmids were transformed into *E. coli* BL21 (DE3) cells. The protein expression and purification were the same as described above except that 10 mM β-mercaptoethanol was included in buffers A–C in order to prevent oxidation of cysteine side chains.

### Fluorescence Labeling

Labeling of the site-specific cysteine mutants of Skp and OmpC (Skp-K55C-Cy3, Skp-E82C-Cy3, Skp-D128C-Cy3, OmpC-D25C-Cy5, OmpC-L139C-Cy5, and OmpC-D290C-Cy5) was performed by first reducing cysteine residues with 10-fold molar excess of tris (2-carboxyethyl) phosphine (TCEP) in 50 mM PBS, 100 mM NaCl (pH 7.0) at 25°C for 30 min. 10-fold molar excess of Cy3-maleimide or Cy5-maleimide (GE Healthcare) was then added to Skp or OmpC protein solution at 100 µM concentration respectively, and the solution was kept in the dark at 25°C for 2 h. Excess dye was removed when the sample was passed over a PD-10 desalting column (GE Healthcare) in 50 mM PBS, 100 mM NaCl (pH 7.0). 8 M urea was included in the labeling and eluting buffer for the labeling of OmpC mutants to prevent its aggregation. For Skp mutants labeled with Cy3, labeling efficiency was hard to be determined by conventional spectrophotometric measurements because three cysteine residues were introduced into one Skp trimer at one time. Dyes labeled on Skp trimer kept so close to each other that their microenvironment was changed, which strongly affected the absorption spectra and extinction coefficients. The method of Meadows et al. was used to determine the correct labeling efficiency as follows [33].

High concentration of guanidinium hydrochloride (GdmCl) was added to the dye/protein solution to give a final 6 M GdmCl solution. The solution was then kept in the dark at 37°C for 1 h to denature protein sufficiently so that Skp trimer could be separated to three monomers and thereby significantly reduce the effects of microenvironment change on the absorbance properties of Cy3. For OmpC mutants labeled with Cy5, the problems mentioned above did not exist because OmpC mutants were kept in the unfolded state in 8 M urea solution. At last the absorbance of these samples at 280 nm (for proteins) and 553 nm (for Cy3) or 650 nm (for Cy5) was used to determine the extent of labeling. For all samples, the extent of labeling was greater than 90%.

### Aggregation Assay

The chaperone activity of Cy3 labeled Skp mutants was verified by its prevention of OmpC aggregation through the light scattering assay carried out on a UV absorbance spectrometer (U3900, HITACHI, Japan) [19]. Unfolded OmpC in 8 M urea was diluted 100× to a final concentration of 1.5 µM using PBS buffer. The buffer contained wild-type Skp of various concentrations (0, 1.5, 4.5 and 7.5 µM, respectively) and 7.5 µM labeled or unlabeled Skp mutants, respectively. The aggregation of OmpC was monitored spectrophotometrically at 380 nm at 25°C. The precipitation of OmpC decreased the transparency of the solution and gave rise to the increased absorbance. Figure 2 shows that 5-fold molar excess of wild-type Skp was least amount necessary to prevent OmpC from aggregation, and the same amount of either unlabeled or Cy3 labeled Skp mutants could work just as well, indicating that the chaperone activity of Skp was maintained after the mutation and labeling.

### CD Measurements

Far-UV CD spectra (200–260 nm) were recorded at 25°C with a Jasco J-815 spectrometer using a 10 mm path length quartz cuvette. The final concentration of labeled and unlabeled proteins in 50 mM sodium phosphate buffer containing 100 mM NaCl (pH 7.0) was 0.1 µM. Averages of 3 scans were used and the final results were obtained by subtraction of blank control from the spectra.

### FCS Measurements

FCS measurements were performed on a home-built dual-channel confocal fluorescence microscope setup that has been described previously [34]. In the current study, excitation of Cy3 dye was accomplished by a 532 nm CW Ya-Ge laser (SUW Tech., China) and the excitation power was adjusted to 200 µW before entering the microscope. Cy3 labeled OmpC in 8 M urea was diluted dropwise in a buffer (50 mM PBS, pH 7.0, 100 mM NaCl) containing 0.2 µM reduced Skp mutants. The ∼40 µL sample solution was sealed between a chamber cover (GraceBio, Sigma, Germany) and a cover glass. Glass surface absorption of proteins was suppressed by adding 0.01% Tween 20 (Sigma, Germany) into the solution buffer. The accumulated measurement time for each FCS curve was 30 min. The FCS data were fitted to a model for diffusion of a single species in two dimensions with exponential relaxations:
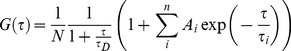
(1)where *τ* is the lag time, *N* is the average number of molecules in the detection confocal volumn, 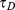 is the translational diffusion time constant, and 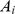
and 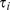
are the respective amplitude and time constant of the dynamic component *i*. For a two- and a three-exponential model, *n* is 2 and 3. Using Cy3 dye as a reference, the molecular weights of Skp-OmpC complexes could be calculated through [35]:
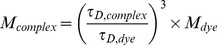
(2)


### Fluorescence Spectroscopy

Fluorescence spectra were recorded on a Shimadzu RF-5301 PC spectrofluorometer. The excitation wavelength was 532 nm, and the bandwidths of the excitation and emission monochromators were both 10 nm. The integration time was 0.05 s, and an increment of 0.2 nm was used to scan spectra in the range of 545–600 nm. All experiments were performed in 50 mM PBS (pH 7.0), 100 mM NaCl, 0.01% Tween 20 (Sigma, Germany) buffer at 25°C. OmpC, OmpA-TMD, or OmpF in 8 M urea was diluted dropwise to PBS buffer containing various Cy3 labeled Skp mutants respectively. The final concentrations of these outer membrane proteins and the corresponding labeled Skp mutants were all 0.03 µM. Samples were kept in the dark at 25°C for 10 min before adding into the cuvette. The reported results were average of three scans.

### Subunit Exchange Experiments

Subunit exchange experiments were carried out by mixing Skp-E82C-Cy3 with 5-fold molar excess of unlabeled Skp-wt at 37°C in 50 mM PBS buffer at pH 7.0. Fluorescence emission of Cy3 dye was monitored in the range of 545–600 nm with an interval of 20 min. The exchange of subunits between labeled Skp mutants and unlabeled wild-type Skp reduced self-quenching and thus led to an increase of fluorescence intensity.

### Fluorescence Stopped-flow Measurements

All fluorescence stopped-flow measurements were performed using a SFM-300 stopped-flow module (Bio-logic, France) equipped with a 532 nm CW Ya-Ge laser (SUW Tech., China) as the light source. The stopped-flow unit and the observation cell with a 1.5 mm path length were thermostated by circulating water from a temperature-controlled bath. The dead time of the instrument was estimated to be 2.4 ms. For Cy3 self-quenching experiments, the Cy3 dye was excited at 532 nm and the fluorescence emission was detected using a 595/50 filter (Semrock, USA). For Cy3 and Cy5 FRET experiments, the Cy3 dye was excited at 532 nm and the Cy5 dye emission was detected using a 685/40 filter (Semrock, USA). All stopped-flow experiments were carried out in 50 mM PBS (pH 7.0), 100 mM NaCl, 0.01% Tween 20 (Sigma, Germany) buffer at 25°C.

To measure the Cy3 self-quenching kinetics, OmpC, OmpA-TMD, or OmpF in 8 M urea solution was mixed with the corresponding Cy3 labeled Skp mutants to a volume ratio of 1∶20 to dilute the urea, achieving a non-denaturing environment. The final concentrations of Cy3 labeled Skp mutants and wild-type OMPs were all 0.03 µM. For the FRET experiments, Cy5 labeled OmpC mutants in 8 M urea solution were mixed with Cy3 labeled Skp mutants with a volume ratio of 1∶20 to achieve a non-denaturing environment. The final concentrations of labeled OmpC mutants and Skp mutants were all 0.03 µM. The kinetic traces represented an average of seven to eight individual scans and were fitted to exponential functions.

### Simulation Details

Present simulation systems include Skp in water, and Skp and various fragments taken from different OMPs in water. Peptides include the N-terminal fragments from OmpC (sequence: AEVYNKDGNKLDLYGKVDGL), OmpA (sequence: APKDNTWYTGAKLGFSQYHDT), and OmpF (sequence: AEIYNKDGNKVDLYGKAVGL), and the C-terminal fragment from OmpC (sequence: FTRDAGINTDNIVALGLVYQF). All MD simulations were performed in explicit solvent making use of AMBER 9.0 suite of programs [36] with FF99 force field [37]. Water was described with SPC/E model [38]. The initial atomic coordinates of Skp was taken from the crystal structure (PDB code: 1SG2 [21]). The thirty-four missing resides (from Glu53 to Phe86) in chain B of Skp were added following the alignment to chain A and C. Each system was prepared by immersing Skp and the corresponding peptide into a cubic box containing water molecules. Cl- anions were added to neutralize the charge of the system. The size of the box and the number of water molecules and Cl- anions in each system are organized in Tables S3 and S4.

For each system, the simulation protocol included the energy minimization, the following heating-up process, and the final longtime equilibrium simulation calculation (production). NPT (constant number, pressure, temperature) ensemble calculations were performed and the periodic boundary conditions were used in the simulations. The SHAKE algorithm [39] was used to constrain all bonds involving hydrogens. A cutoff of 8.0 Å was applied for nonbonding interactions. The Particle Mesh Ewald method was applied to treat long-range electrostatic interactions [40].

Initially, the energies of the systems were minimized through a total of 2500 steps of calculations: the first 1000 steps were the steepest descent minimization with the polypeptide being fixed using harmonic restraints, with a force constant of 500.0 kcal mol^−1 ^Å^−2^ applied to the backbone atoms, the following 1500 steps were the conjugate gradient minimization. Subsequently, the systems were heated to 360 K within 200 ps and equilibrated at 360 K for 1 ns, followed by a 200 ps cooling from 360 to 300 K (these heating, equilibration and cooling processes were run with harmonic restraints applied to the backbone atoms (force constant = 10.0 kcal mol^−1^ Å^−2^); the equilibration at high temperature made the polypeptide and Skp dissolved in water). Finally, the production runs were performed and then used for the data analysis, with the data being collected every 1.0 ps.

## Supporting Information

Figure S1 **Far-UV CD spectra of wild-type Skp and fluorescence labeled and unlabeled Skp mutants.** Spectra of 0.1 µM proteins were collected in 50 mM PBS, 100 mM NaCl (pH 7.0) using a 10 mm path length quartz cuvette.
(TIF)

Figure S2 **FCS curves for individual Cy3 dye and Cy3 labeled OmpC binding with Skp mutants.** These data have been offset for clarity. Red lines represent the global fits of these data to Eq. (1) with *n* = 2 for individual Cy3 dye and *n* = 3 for the others.
(TIF)

Figure S3 **Binding of OmpA-TMD with Cy3 labeled Skp mutants measured by stopped-flow kinetics experiment.** (A*–*C) Fluorescence time-course monitoring the entrance of OmpA-TMD into the Cy3 dye labeled Skp mutants (Skp-K55C-Cy3, Skp-E82C-Cy3, and Skp-D128C-Cy3). The increase in intrinsic fluorescence of Cy3 dye labeled at the three sites of Skp was used to monitor the entrance. The data were fitted with two or three exponentials. (D) Comparison of the static fluorescence intensity of Cy3 dye labeled Skp mutants in the presence and absence of OmpA-TMD.
(TIF)

Figure S4 **Binding of OmpF with Cy3 labeled Skp mutants measured by stopped-flow kinetics experiment.** (A*–*C) Fluorescence time-course monitoring the entrance of OmpF into the Cy3 dye labeled Skp mutants (Skp-K55C-Cy3, Skp-E82C-Cy3, and Skp-D128C-Cy3). The increase in intrinsic fluorescence of Cy3 dye labeled at the three sites of Skp was used to monitor the entrance. The data were fitted with two or three exponentials. (D) Comparison of the static fluorescence intensity of Cy3 dye labeled Skp mutants in the presence and absence of OmpF.
(TIF)

Figure S5 **The choice on fitting functions for fluorescence time-course of interactions between Skp-Cy3 and OmpC-Cy5.** The fitting residues with double exponentials or triple exponentials demonstrate that triple exponentials are needed for a satisfactory fitting.
(TIF)

Figure S6 **Snapshots from the trajectory of the N-terminal polypeptide of OmpA in the bottom of open Skp.** Salt bridges formed between the polypeptide and Skp are shown with the dashed lines and residues involved are shown with the licorice mode. For each Skp residue, the corresponding chain is recorded in the parentheses.
(TIF)

Figure S7 **Snapshots from the trajectory of the N-terminal polypeptide of OmpF in the bottom of open Skp.** Salt bridges formed between the polypeptide and Skp are shown with the dashed lines and residues involved are shown with the licorice mode. For each Skp residue, the corresponding chain is given in the parentheses.
(TIF)

Figure S8 **Time evolution of the distance between the first residue of the N-terminal polypeptide of OmpA (OmpC) and E82 of chain A in Skp.** Three simulation trajectories with the polypeptide placed at the side of Skp are shown. The initial position of the polypeptide relative to Skp is shown in the left panel. The first residue of the polypeptide and E82 in Skp are shown with the VDW mode.
(TIF)

Figure S9 **Negative control simulations showing that the uncharged Asn (Ala) mutated N-terminal fragment of OmpC fails to enter Skp.** (A) Time evolution of the distance in the z-direction between Lys55 of Skp and the N-terminal residue (NT) or C-terminal residue (CT) of the polypeptide when the open state of Skp was used in simulation. In the case of the Asn mutant, although at the end of simulation the C-terminus was at the same level of the Skp bottom, the entire polypeptide actually already moved out of Skp. (B) Time evolution of distances between the Lys55 residues in three chains of Skp.
(TIF)

Figure S10 **Negative control simulations showing that the uncharged Asn (Ala) mutated N-terminal fragment of OmpA fails to enter Skp.** (A) Time evolution of the distance in the z-direction between Lys55 of Skp and the N-terminal residue (NT) or C-terminal residue (CT) of the polypeptide when the open state of Skp is used in simulation. (B) Time evolution of distances between the Lys55 residues in three chains of Skp.
(TIF)

Figure S11 **Time evolution of the z-direction distance between Lys55 of Skp to the N-terminal residue (NT) and the C-terminal residue (CT) of C-terminal fragment of OmpC.** Nine independent trajectories with open state of Skp are shown.
(TIF)

Figure S12 **Time evolution of the salt bridge number formed between the C-terminal polypeptide of OmpC and Skp.** Nine independent trajectories with respect to Figure 11 are analyzed.
(TIF)

Table S1 Summary of protein mutants and PCR primers used in this study.
(PDF)

Table S2 Time constants *τ*_i_ and respective pre-exponential amplitude *A*_i_ for binding of Skp-Cy3 mutants with OmpC-Cy5 obtained from the fitting of the stopped-flow trace in Figure 5.
(PDF)

Table S3 Parameters used in simulations for Skp and N-terminal fragments of OmpC, OmpA, and OmpF in water, respectively.
(PDF)

Table S4 Parameters for nine simulations of Skp and the C-terminal fragment of OmpC in water.
(PDF)

Table S5 The characteristics of polypeptides in molecular dynamics simulations.
(PDF)
