# Supplementary figures and images for: Direct Observation of the Uptake of Outer Membrane Proteins by the Periplasmic Chaperone Skp
Source: PLoS One. 2012 Sep 26;7(9):e46068. doi: 10.1371/journal.pone.0046068 (PMC3458824; doi:10.1371/journal.pone.0046068)

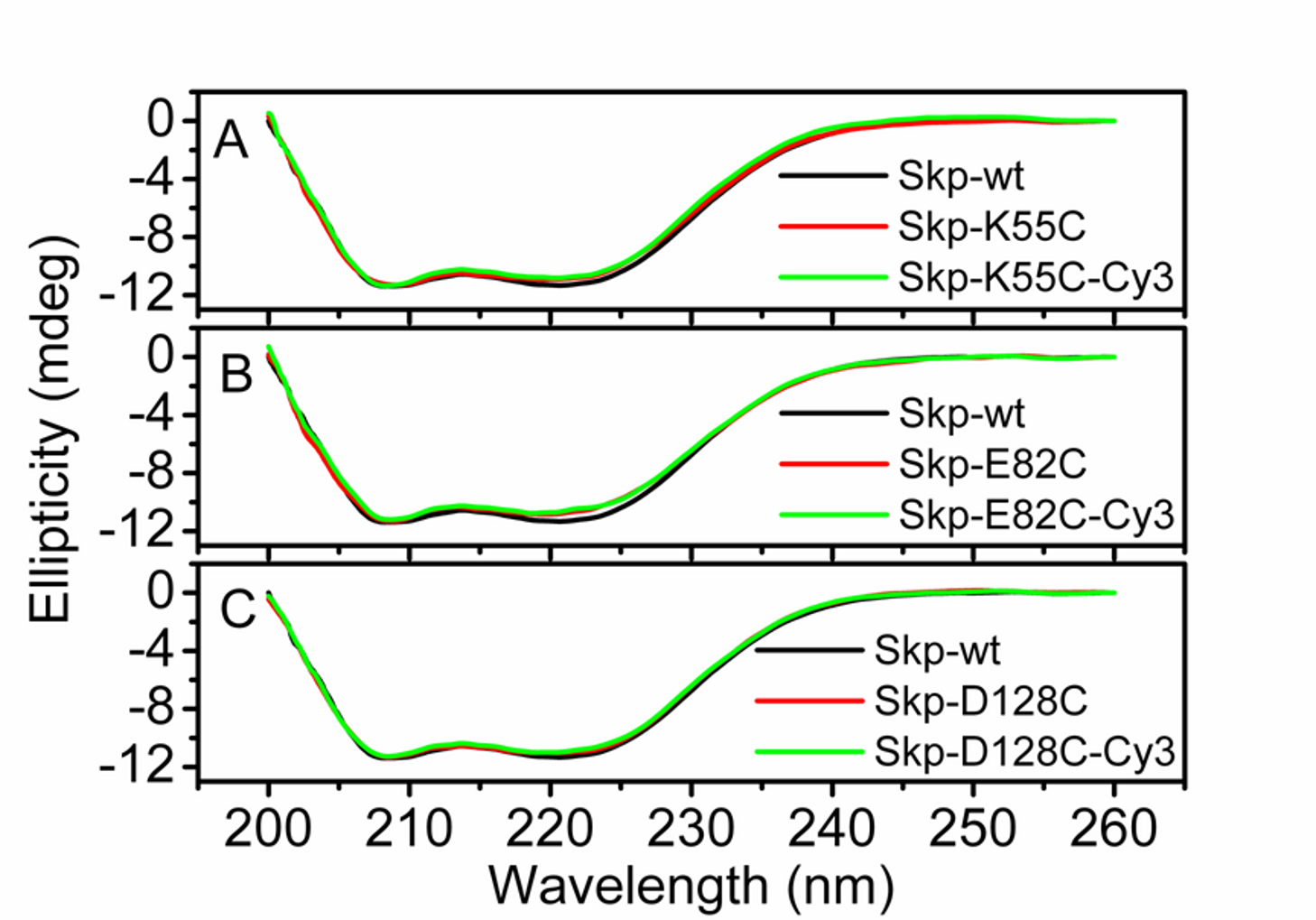

Supplement: Figure S1 — Far-UV CD spectra of wild-type Skp and fluorescence labeled and unlabeled Skp mutants. Spectra of 0.1 µM proteins were collected in 50 mM PBS, 100 mM NaCl (pH 7.0) using a 10 mm path length quartz cuvette. (TIF) [file pone.0046068.s001.tif]

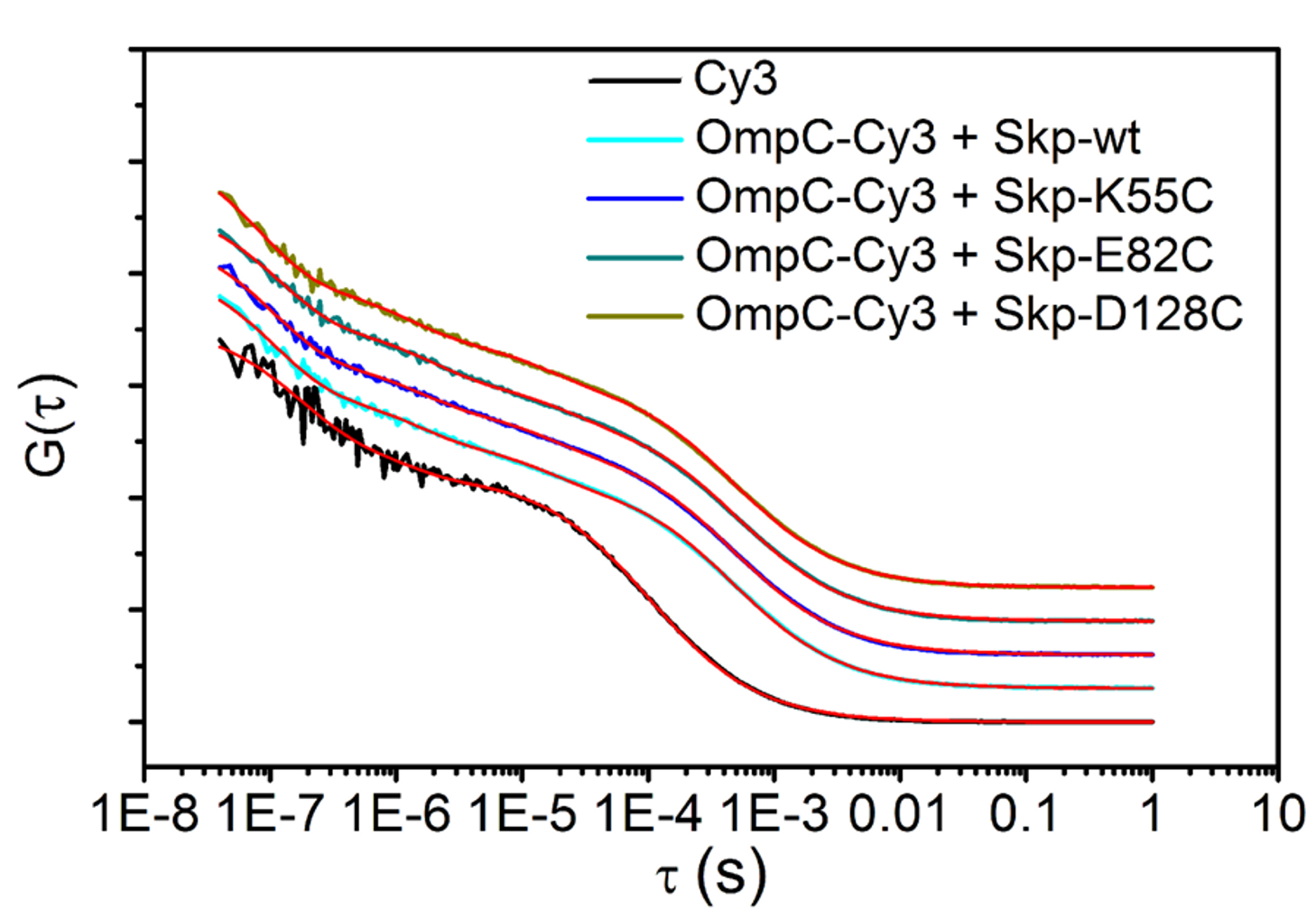

Supplement: Figure S2 — FCS curves for individual Cy3 dye and Cy3 labeled OmpC binding with Skp mutants. These data have been offset for clarity. Red lines represent the global fits of these data to Eq. (1) with n = 2 for individual Cy3 dye and n = 3 for the others. (TIF) [file pone.0046068.s002.tif]

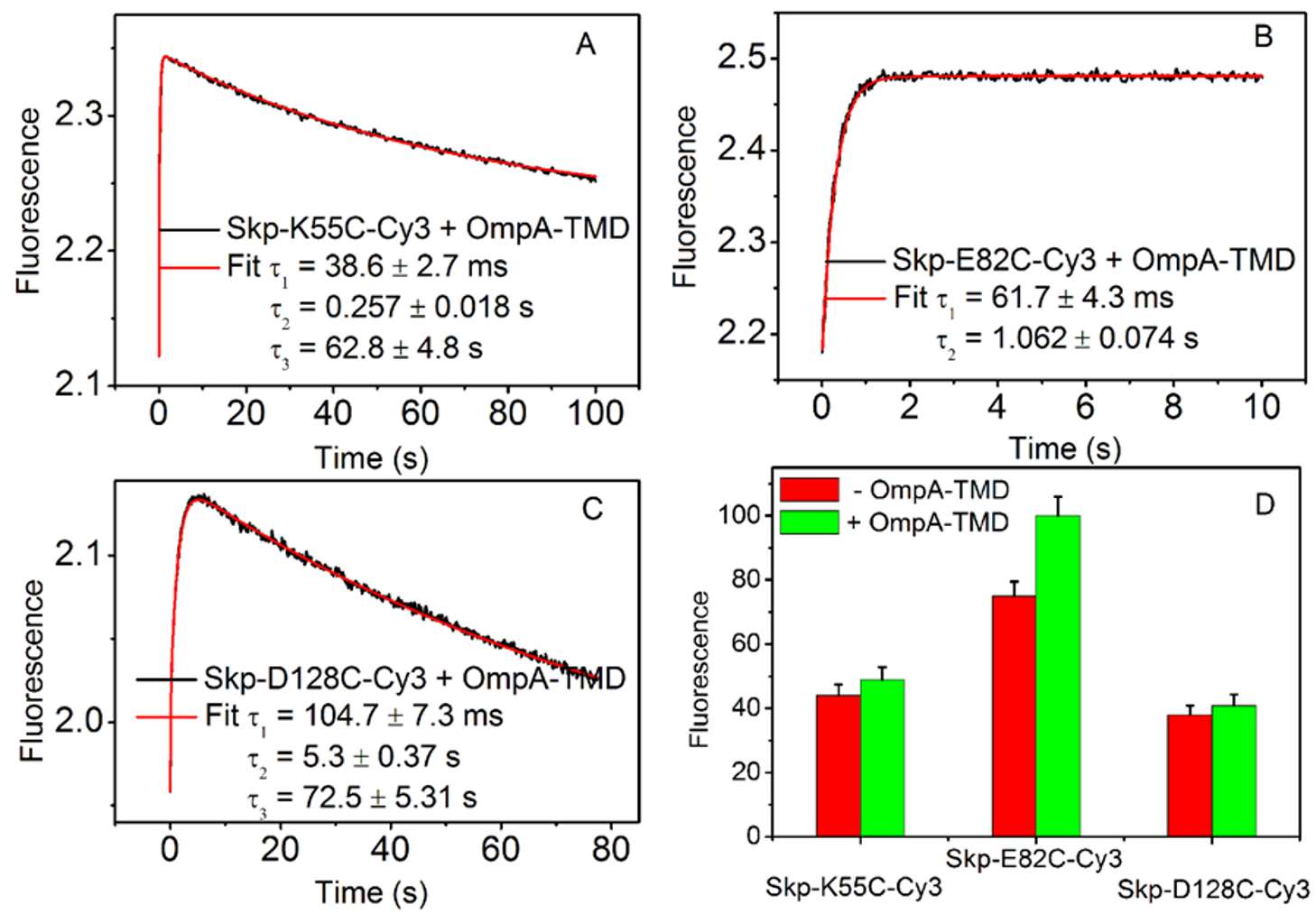

Supplement: Figure S3 — Binding of OmpA-TMD with Cy3 labeled Skp mutants measured by stopped-flow kinetics experiment. (A–C) Fluorescence time-course monitoring the entrance of OmpA-TMD into the Cy3 dye labeled Skp mutants (Skp-K55C-Cy3, Skp-E82C-Cy3, and Skp-D128C-Cy3). The increase in intrinsic fluorescence of Cy3 dye labeled at the three sites of Skp was used to monitor the entrance. The data were fitted with two or three exponentials. (D) Comparison of the static fluorescence intensity of Cy3 dye labeled Skp mutants in the presence and absence of OmpA-TMD. (TIF) [file pone.0046068.s003.tif]

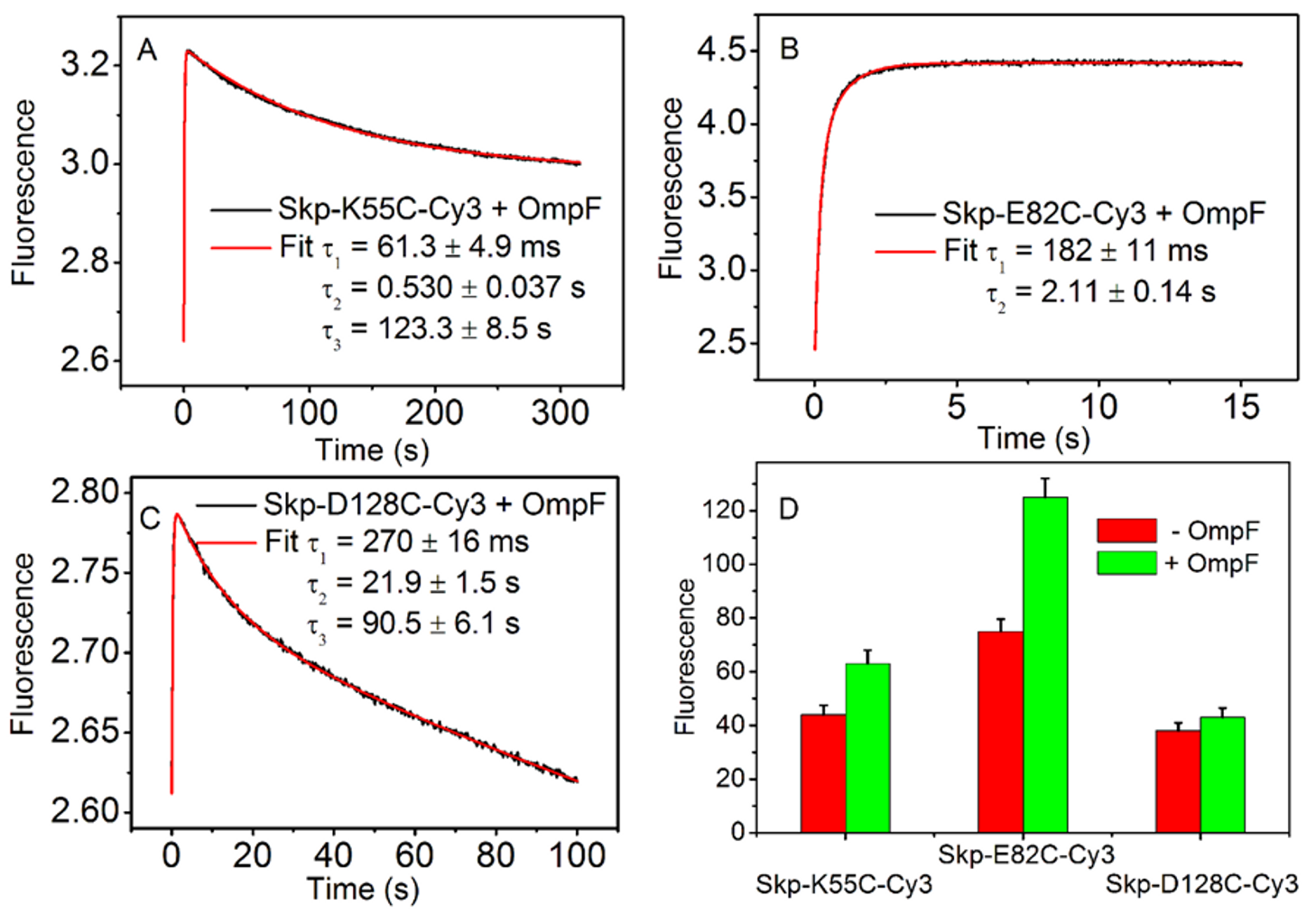

Supplement: Figure S4 — Binding of OmpF with Cy3 labeled Skp mutants measured by stopped-flow kinetics experiment. (A–C) Fluorescence time-course monitoring the entrance of OmpF into the Cy3 dye labeled Skp mutants (Skp-K55C-Cy3, Skp-E82C-Cy3, and Skp-D128C-Cy3). The increase in intrinsic fluorescence of Cy3 dye labeled at the three sites of Skp was used to monitor the entrance. The data were fitted with two or three exponentials. (D) Comparison of the static fluorescence intensity of Cy3 dye labeled Skp mutants in the presence and absence of OmpF. (TIF) [file pone.0046068.s004.tif]

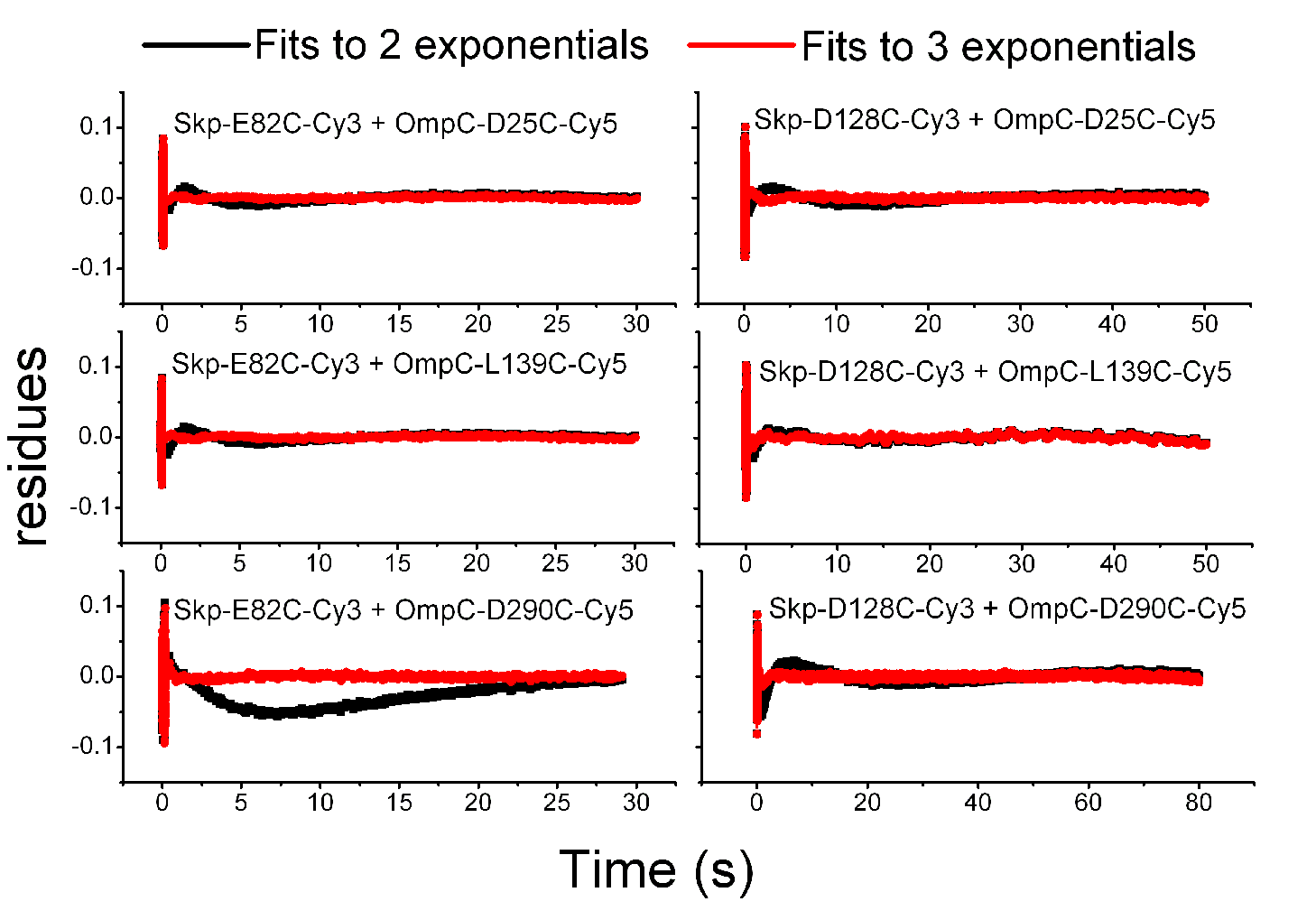

Supplement: Figure S5 — The choice on fitting functions for fluorescence time-course of interactions between Skp-Cy3 and OmpC-Cy5. The fitting residues with double exponentials or triple exponentials demonstrate that triple exponentials are needed for a satisfactory fitting. (TIF) [file pone.0046068.s005.tif]

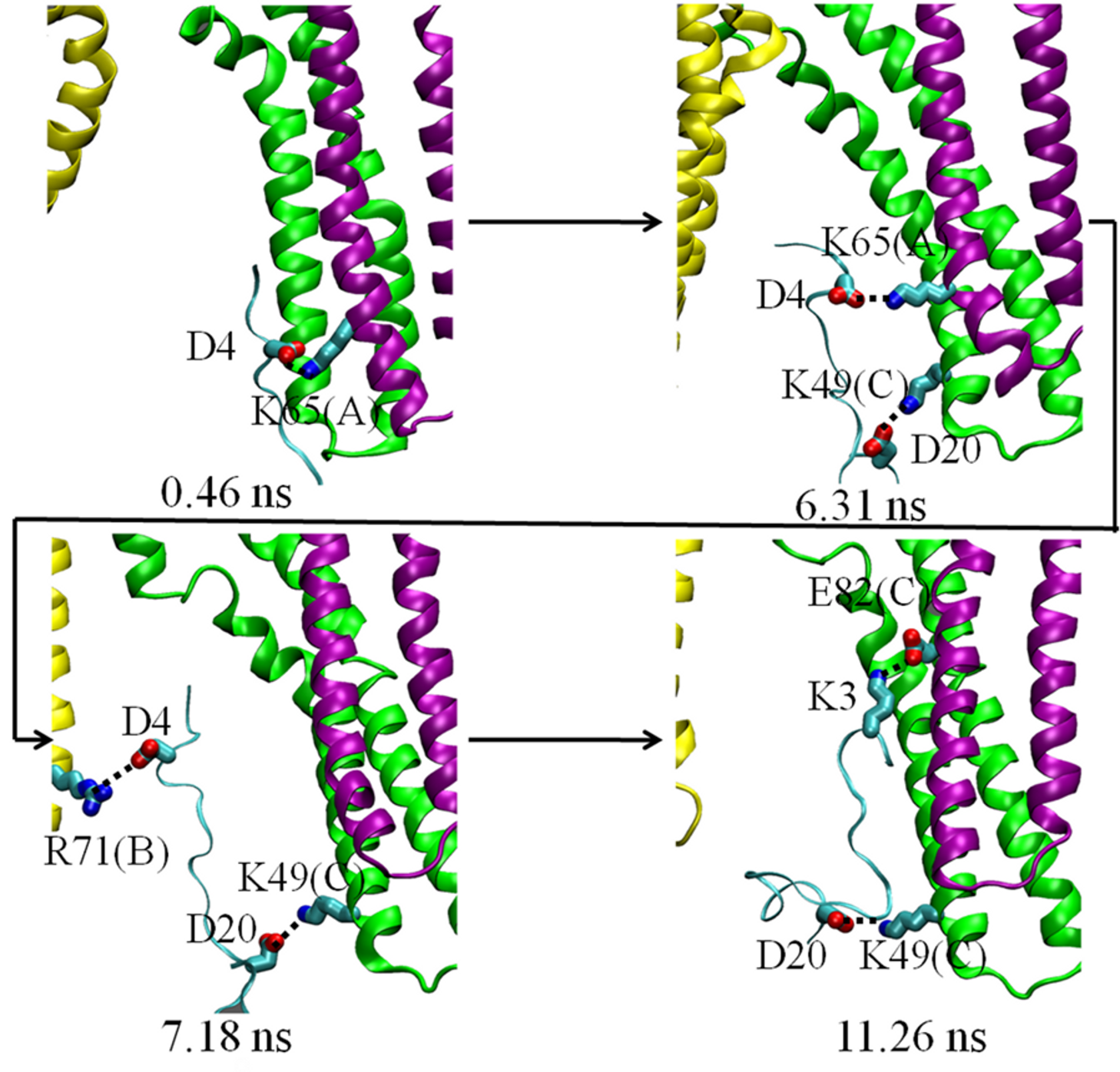

Supplement: Figure S6 — Snapshots from the trajectory of the N-terminal polypeptide of OmpA in the bottom of open Skp. Salt bridges formed between the polypeptide and Skp are shown with the dashed lines and residues involved are shown with the licorice mode. For each Skp residue, the corresponding chain is recorded in the parentheses. (TIF) [file pone.0046068.s006.tif]

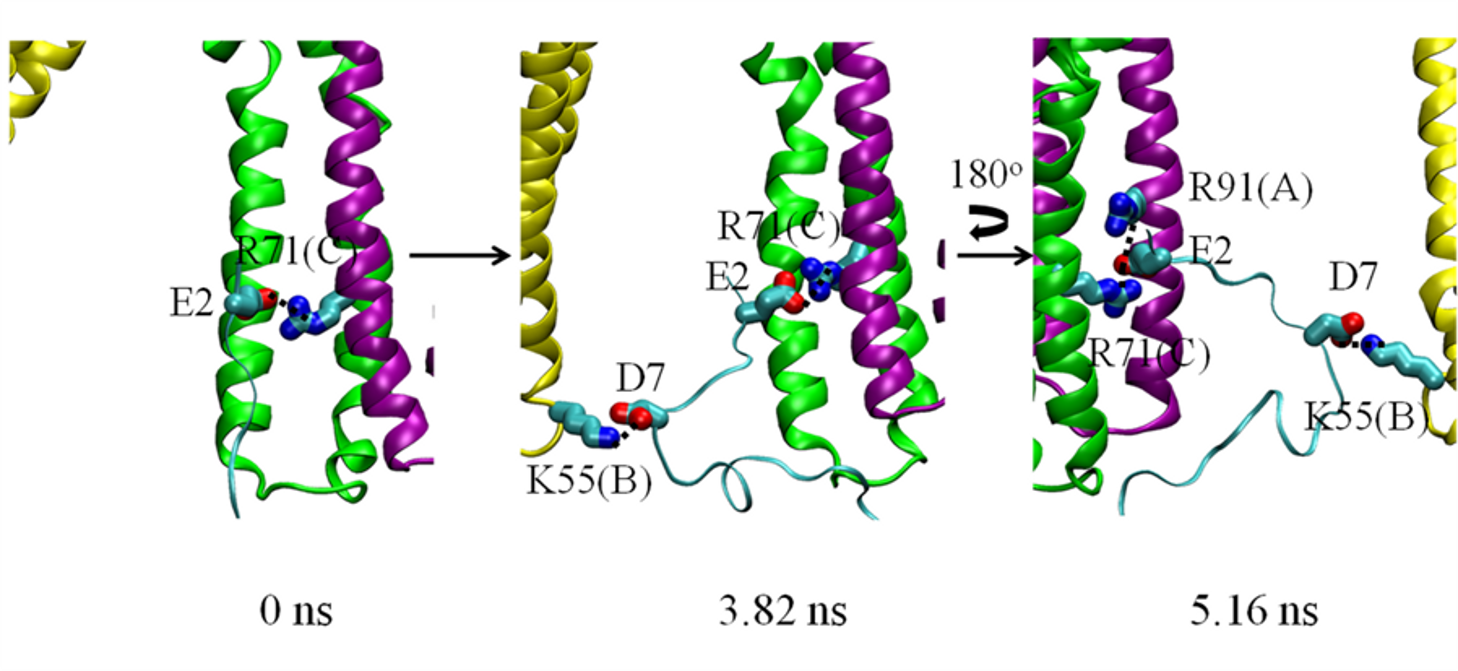

Supplement: Figure S7 — Snapshots from the trajectory of the N-terminal polypeptide of OmpF in the bottom of open Skp. Salt bridges formed between the polypeptide and Skp are shown with the dashed lines and residues involved are shown with the licorice mode. For each Skp residue, the corresponding chain is given in the parentheses. (TIF) [file pone.0046068.s007.tif]

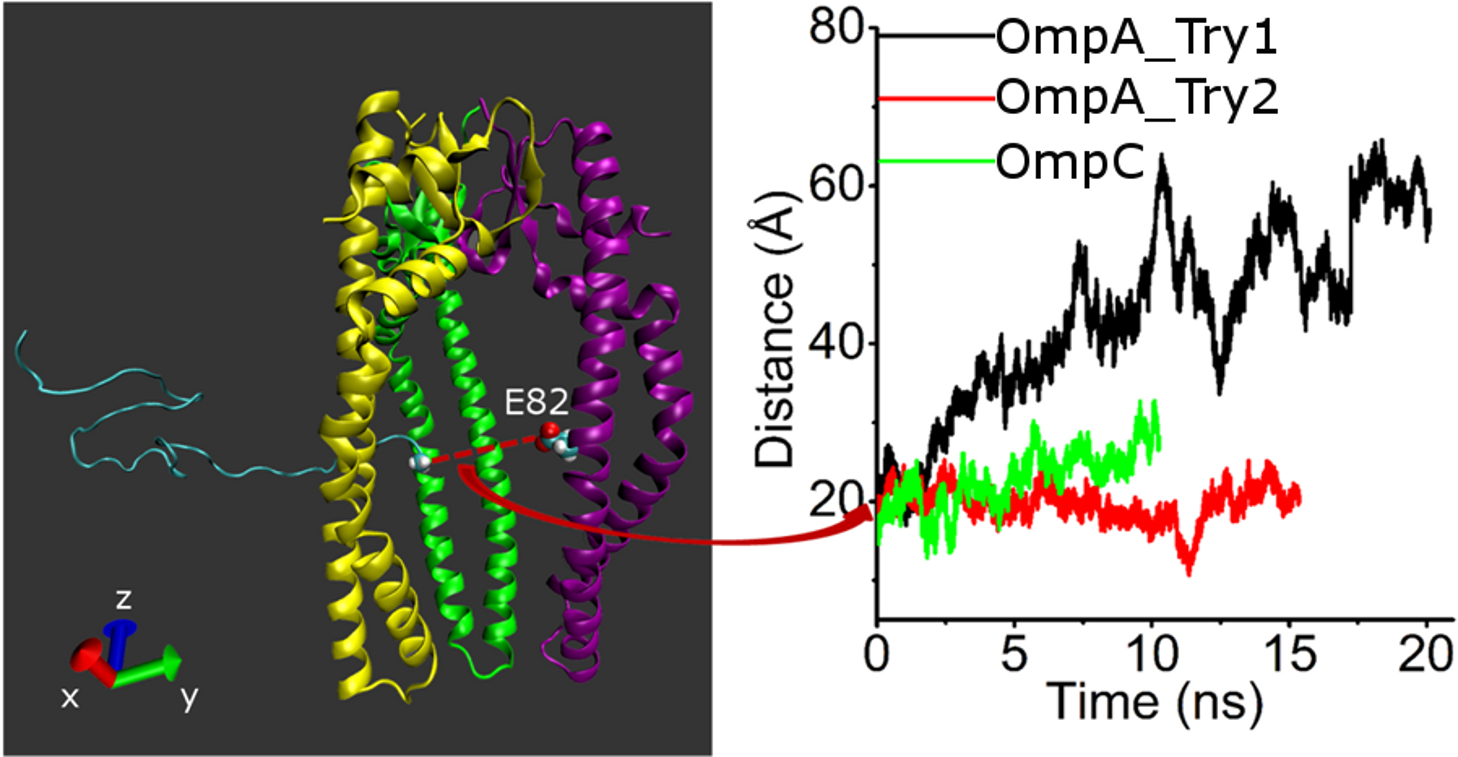

Supplement: Figure S8 — Time evolution of the distance between the first residue of the N-terminal polypeptide of OmpA (OmpC) and E82 of chain A in Skp. Three simulation trajectories with the polypeptide placed at the side of Skp are shown. The initial position of the polypeptide relative to Skp is shown in the left panel. The first residue of the polypeptide and E82 in Skp are shown with the VDW mode. (TIF) [file pone.0046068.s008.tif]

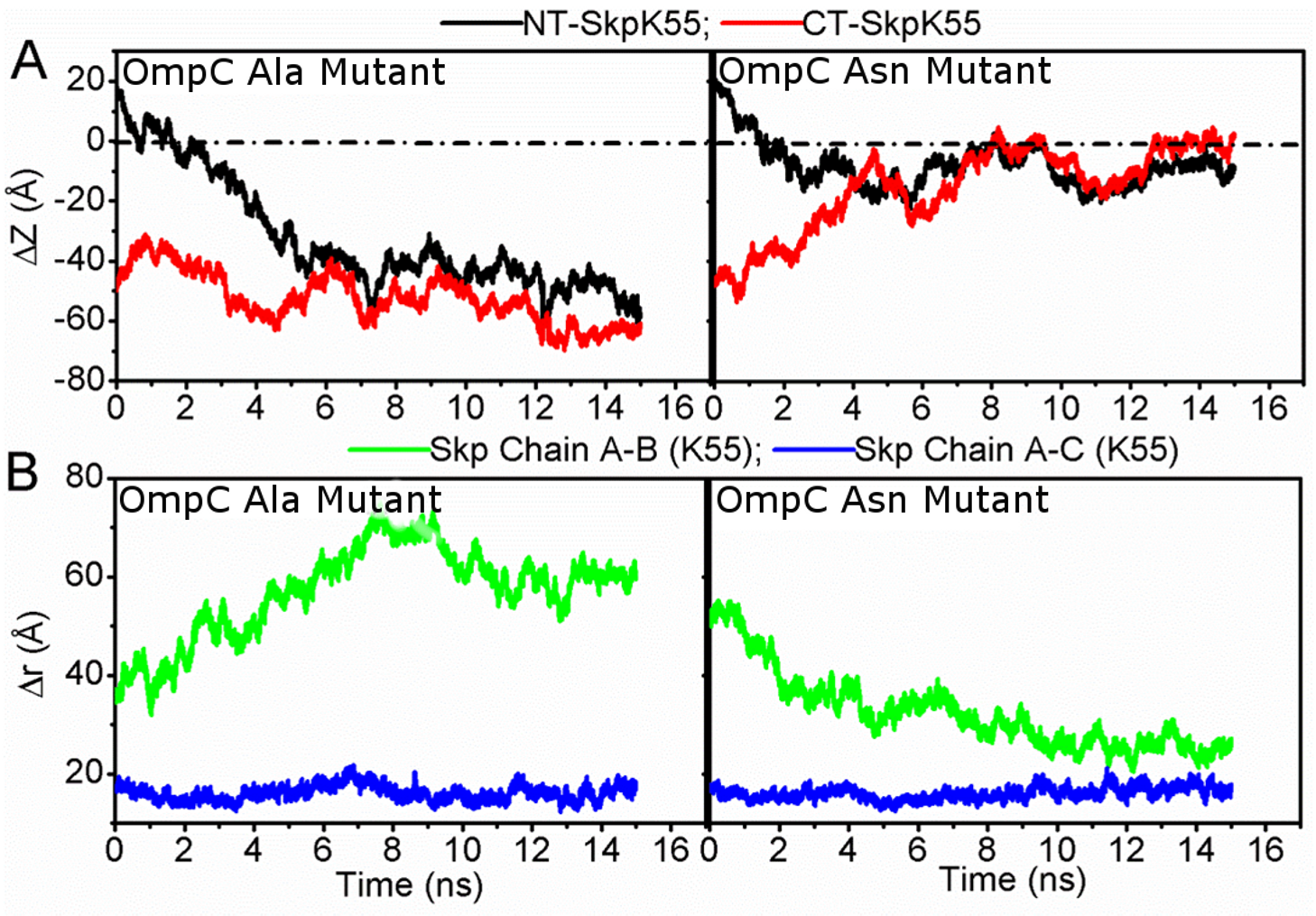

Supplement: Figure S9 — Negative control simulations showing that the uncharged Asn (Ala) mutated N-terminal fragment of OmpC fails to enter Skp. (A) Time evolution of the distance in the z-direction between Lys55 of Skp and the N-terminal residue (NT) or C-terminal residue (CT) of the polypeptide when the open state of Skp was used in simulation. In the case of the Asn mutant, although at the end of simulation the C-terminus was at the same level of the Skp bottom, the entire polypeptide actually already moved out of Skp. (B) Time evolution of distances between the Lys55 residues in three chains of Skp. (TIF) [file pone.0046068.s009.tif]

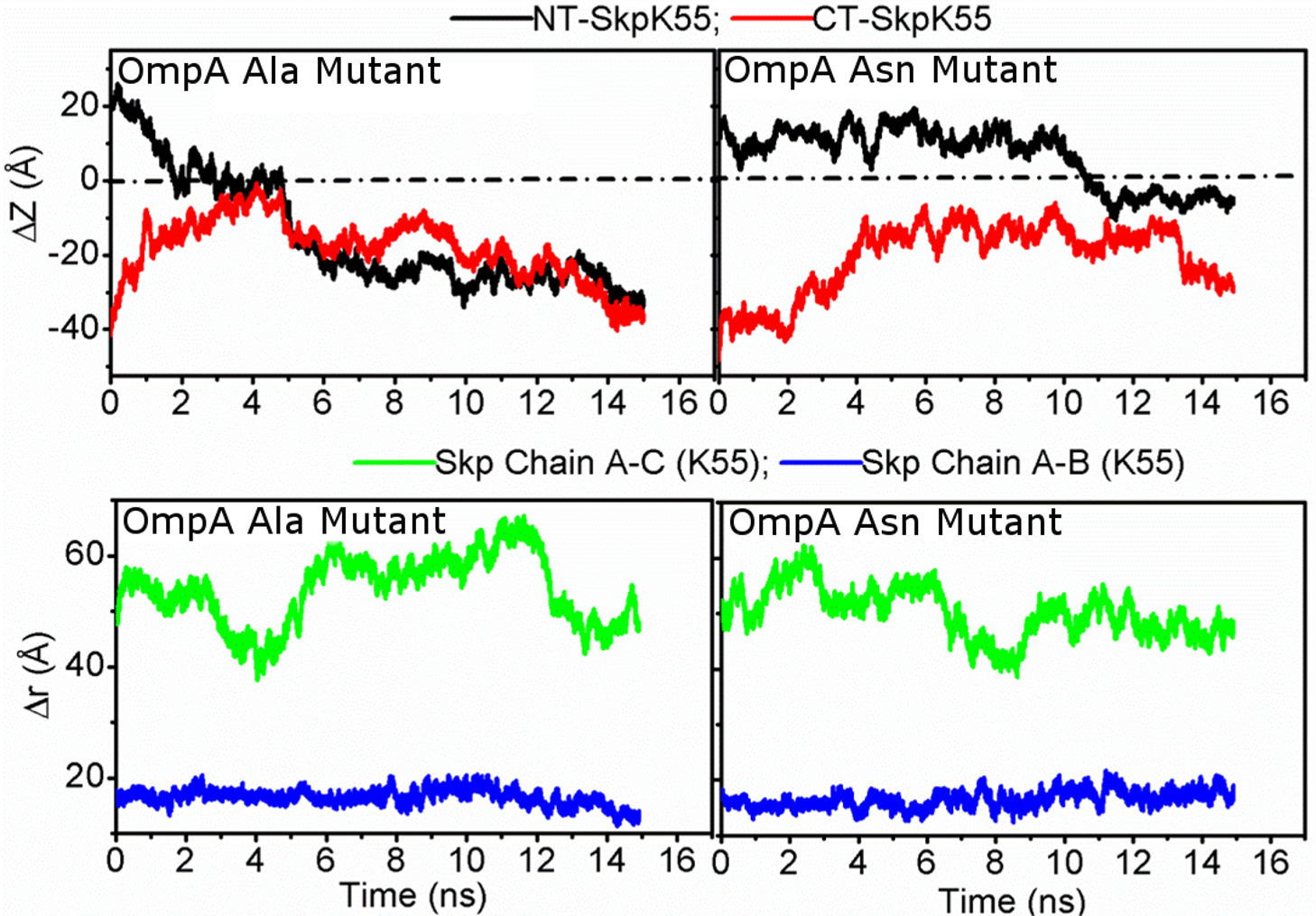

Supplement: Figure S10 — Negative control simulations showing that the uncharged Asn (Ala) mutated N-terminal fragment of OmpA fails to enter Skp. (A) Time evolution of the distance in the z-direction between Lys55 of Skp and the N-terminal residue (NT) or C-terminal residue (CT) of the polypeptide when the open state of Skp is used in simulation. (B) Time evolution of distances between the Lys55 residues in three chains of Skp. (TIF) [file pone.0046068.s010.tif]

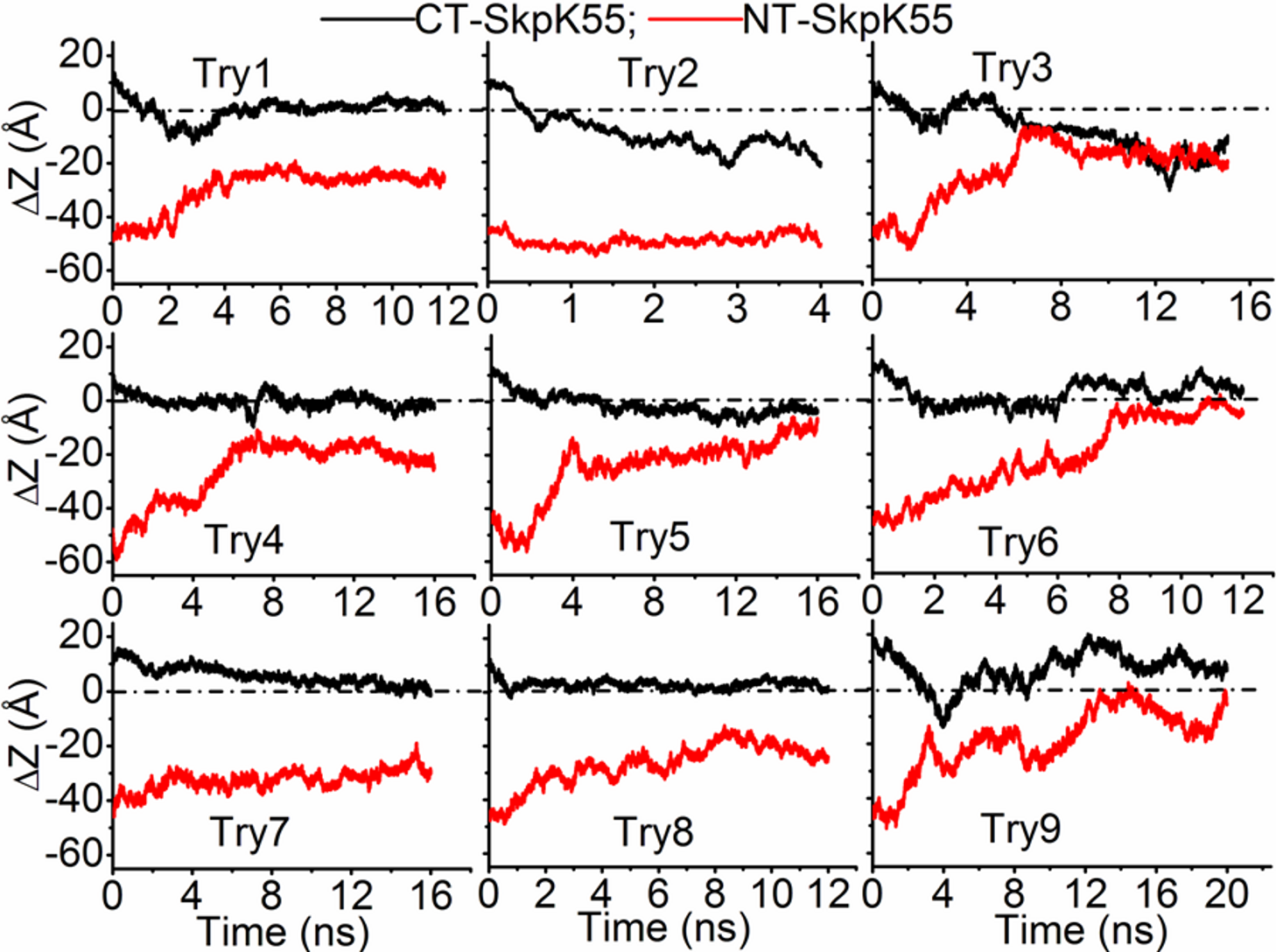

Supplement: Figure S11 — Time evolution of the z-direction distance between Lys55 of Skp to the N-terminal residue (NT) and the C-terminal residue (CT) of C-terminal fragment of OmpC. Nine independent trajectories with open state of Skp are shown. (TIF) [file pone.0046068.s011.tif]

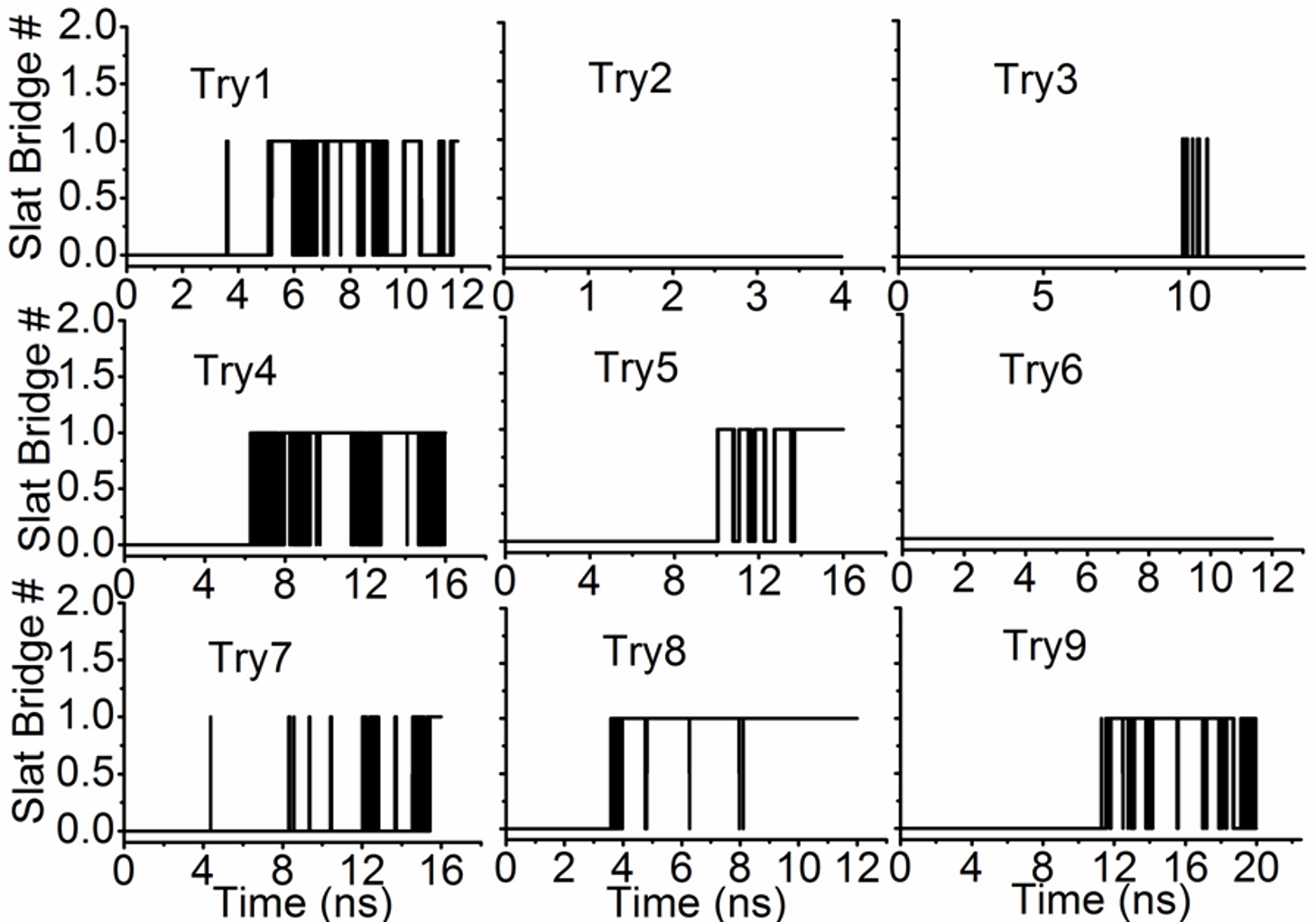

Supplement: Figure S12 — Time evolution of the salt bridge number formed between the C-terminal polypeptide of OmpC and Skp. Nine independent trajectories with respect to Figure 11 are analyzed. (TIF) [file pone.0046068.s012.tif]
