# Supplementary material for: Direct Observation of the Uptake of Outer Membrane Proteins by the Periplasmic Chaperone Skp
Source: PLoS One. 2012 Sep 26;7(9):e46068. doi: 10.1371/journal.pone.0046068 (PMC3458824; doi:10.1371/journal.pone.0046068)
Supplement: Table S1 — Summary of protein mutants and PCR primers used in this study. (PDF) [file pone.0046068.s013.pdf]

Table S1 Summary of protein mutants and PCR primers used in this study *

Protein mutants	Primer	DNA sequences (5'-3')
Skp-K55C	Forward	GCAGTCCATGTGCGCGGGCAGCG
	Reverse	CGCTGCCCGCGCACATGGACTGC
Skp-E82C	Forward	AAGCGCAGGCTTTTTGCCAGGATCGCGCACG
	Reverse	AAAAGCCTGCGCTTTCTGAGCAAAAGTCTGG
Skp-D128C	Forward	CAACAGCAGCTGTGTAAAAGACATCACTGCCG
	Reverse	GATGTCTTTTACACAGCTGCTGTTGTAAGCAAC
OmpC-D25C	Forward	CACTATTTCTCTTGTAACAAAGATGTAGATGGCG
	Reverse	CTACATCTTTGTTACAAGAGAAATAGTGCAGGC
OmpC-L139C	Forward	CTTCTTCGGTTGTGTTGACGGCCTGAACTTTG
	Reverse	GCCGTCAACACAACCGAAGAAGTCAGTG
OmpC-D290C	Forward	GTCGTGGCTACTGCGACGAAGATATCCTG
	Reverse	ATATCTTCGTCGCAGTAGCCACGACCCAG

* A pair of forward and reverse primers was used to create each mutant. Changes in the sequences were shown underlined.
