# Supplementary material for: Direct Observation of the Uptake of Outer Membrane Proteins by the Periplasmic Chaperone Skp
Source: PLoS One. 2012 Sep 26;7(9):e46068. doi: 10.1371/journal.pone.0046068 (PMC3458824; doi:10.1371/journal.pone.0046068)
Supplement: Table S2 — Time constants τ i and respective pre-exponential amplitude A i for binding of Skp-Cy3 mutants with OmpC-Cy5 obtained from the fitting of the stopped-flow trace in Figure 5. (PDF) [file pone.0046068.s014.pdf]

Table S2 Time constants τ_i and respective pre-exponential amplitude A_i for binding of Skp-Cy3mutants with OmpC-Cy5 obtained from the fitting of the stopped-flow data.

		Skp-K55C-Cy3	Skp-E82C-Cy3	Skp-D128C-Cy3
OmpC-D25C-Cy5	τ_1	73.0 ± 5.1 ms	101.3 ± 6.9 ms	143 ± 10 ms
	A_1	0.655 ± 0.078	0.573 ± 0.069	0.609 ± 0.073
	τ_2	0.264 ± 0.018 s	0.437 ± 0.030 s	0.83 ± 0.056 s
	A_2	0.329 ± 0.039	0.303 ± 0.032	0.297 ± 0.035
	τ_3	40.5 ± 2.7 s	2.58 ± 0.18 s	6.88 ± 0.46 s
	A_3	-(0.181 ± 0.022)	0.142 ± 0.015	0.174 ± 0.020
OmpC-L139C-Cy5	τ_1	86.3 ± 6.0 ms	135.2 ± 9.3 ms	208 ± 14 ms
	A_1	0.456 ± 0.055	0.506 ± 0.061	0.675 ± 0.081
	τ_2	0.325 ± 0.023 s	0.53 ± 0.037 s	0.94 ± 0.066 s
	A_2	0.299 ± 0.036	0.271 ± 0.032	0.283 ± 0.035
	τ_3	41.2 ± 2.8 s	2.89 ± 0.20 s	6.95 ± 0.47 s
	A_3	-(0.140 ± 0.017)	0.151 ± 0.018	0.183 ± 0.021
OmpC-D290C-Cy5	τ_1	115.0 ± 7.9 ms	180 ± 13 ms	285 ± 19 ms
	A_1	0.503 ± 0.060	0.572 ± 0.069	0.641 ± 0.077
	τ_2	0.65 ± 0.045 s	0.96 ± 0.066 s	1.52 ± 0.10 s
	A_2	0.305 ± 0.036	0.282 ± 0.033	0.278 ± 0.033
	τ_3	69.2 ± 4.7 s	4.29 ± 0.31 s	9.33 ± 0.64 s
	A_3	-(0.185 ± 0.022)	0.146 ± 0.017	0.187 ± 0.025
