# Supplementary material for: Direct Observation of the Uptake of Outer Membrane Proteins by the Periplasmic Chaperone Skp
Source: PLoS One. 2012 Sep 26;7(9):e46068. doi: 10.1371/journal.pone.0046068 (PMC3458824; doi:10.1371/journal.pone.0046068)
Supplement: Table S3 — Parameters used in simulations for Skp and N-terminal fragments of OmpC, OmpA, and OmpF in water, respectively. (PDF) [file pone.0046068.s015.pdf]

Table S3 Parameters used in simulations for Skp and N-terminal fragments of OmpC, OmpA, and OmpF in water, respectively.

Skp State	Polypeptide	Water	Cl-	Simulation Length
Close	------	16639	15	18.6 ns
Close	N-terminal Fragment of OmpC	22589	14	13.1 ns
Close	N-terminal Fragment of OmpC	27125	14	15.5 ns
Close	N-terminal Fragment of OmpC	27125	14	16.4 ns
Open	N-terminal Fragment of OmpC	28230	14	39.0 ns
Open	N-terminal Fragment of OmpA	27116	15	13.2 ns
Open	N-terminal Fragment of OmpF	27256	15	5.0 ns
