# Supplementary material for: Direct Observation of the Uptake of Outer Membrane Proteins by the Periplasmic Chaperone Skp
Source: PLoS One. 2012 Sep 26;7(9):e46068. doi: 10.1371/journal.pone.0046068 (PMC3458824; doi:10.1371/journal.pone.0046068)
Supplement: Table S4 — Parameters for nine simulations of Skp and the C-terminal fragment of OmpC in water. (PDF) [file pone.0046068.s016.pdf]

Table S4 Parameters for nine simulations of Skp and the C-terminal fragment of OmpC in water.

Skp State	Polypeptide		Water	Cl-	Simulation Length
Open	C-terminal Fragment of OmpC	Trial 1	26324	14	11.9 ns
		Trial 2	26329	14	4.0 ns
		Trial 3	26331	14	15.1 ns
		Trial 4	26328	14	16.0 ns
		Trial 5	26327	14	16.0 ns
		Trial 6	26329	15	12.0 ns
		Trial 7	25926	14	16.0 ns
		Trial 8	25916	14	11.2 ns
		Trial 9	25913	14	20.0 ns
