# Supplementary material for: Direct Observation of the Uptake of Outer Membrane Proteins by the Periplasmic Chaperone Skp
Source: PLoS One. 2012 Sep 26;7(9):e46068. doi: 10.1371/journal.pone.0046068 (PMC3458824; doi:10.1371/journal.pone.0046068)
Supplement: Table S5 — The characteristics of polypeptides in molecular dynamics simulations. (PDF) [file pone.0046068.s017.pdf]

Table S5 The characteristics of polypeptides in molecular dynamics simulations.

Polypeptide	Sequence	Number of Hydrophobic Residues	Number of Charged Residues	Length
OmpC N-terminal Fragment	AEVYNKDGNKLDLYGKVDGL	8	7	20
OmpA N-terminal Fragment	APKDNTWYTGAKLGFSQYHDT	7	5	21
OmpF N-terminal Fragment	AEIYNKDGNKVDLYGKAVGL	9	6	20
OmpC C-terminal Fragment	FTRDAGINTDNIVALGLVYQF	11	3	21
OmpC Ala Mutant	Ace-AAVYNAAGNALALYGAVAGL-Nhe	15	0	20
OmpC Asn Mutant	Ace-ANVYNNNGNNLNLYGNVNGL-Nhe	8	0	20
OmpA Ala Mutant	Ace-APAANTWYTGAALGFSQYAAT-Nhe	12	0	21
OmpA Asn Mutant	Ace-APNNNTWYTGANLGFSQYNNT-Nhe	7	0	21
